# The Japanese Macaque as a Diabetes Recipient Animal Model for Porcine Islet Xenotransplantation

**DOI:** 10.1002/mco2.70726

**Published:** 2026-04-08

**Authors:** Naoaki Sakata, Gumpei Yoshimatsu, Ryo Kawakami, Seiichi Tanaka, Shohta Kodama

**Affiliations:** ^1^ Department of Regenerative Medicine and Transplantation Faculty of Medicine Fukuoka University Jonan‐ku Fukuoka Japan; ^2^ Center For Regenerative Medicine Fukuoka University Hospital Jonan‐ku Fukuoka Japan; ^3^ Center For Experimental Animals Fukuoka University Jonan‐ku Fukuoka Japan

**Keywords:** animal model, diabetes, monkey, pig islet transplantation, xenotransplantation

## Abstract

Porcine islet xenotransplantation is effective for severe diabetes; however, preclinical studies are essential. In this study, we evaluated the suitability of the Japanese macaque as a recipient model for islet xenotransplantation, including identifying the preferred method to induce diabetes. The safety and stability of the following four models to induce diabetes were assessed: Model 1: pancreatectomy, Model 2: pancreatectomy with low‐dose streptozotocin (STZ), Model 3: single‐injection of STZ, and Model 4: consecutive administrations of low‐dose STZ. Diabetes was induced in all four models. The blood glucose level after induction of diabetes was 225.32 ± 46.49 mg/dL in Model 1, 209.64 ± 64.36 mg/dL in Model 2, 175.51 ± 45.18 mg/dL in Model 3, and 139.22 ± 6.31 mg/dL in Model 4. Regarding safety, Models 1 and 2 involved invasive surgery with postoperative concerns. Model 3 induced diabetes in the Japanese macaques; however, the preferable dose of STZ was individual dependent. Among the models, Model 4 was preferable regarding safety and stability. Finally, we performed porcine islet xenotransplantation in a diabetic monkey in Model 4 and evaluated the therapeutic effects of this treatment. In conclusion, the Japanese macaque might be a possible recipient model for porcine islet xenotransplantation.

## Graphical abstract

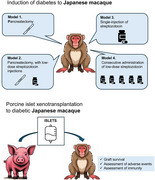

## 1 Introduction

Diabetes mellitus affects an individual's quality of life, may be life threatening, and is characterized by hyperglycemia. In particular, advanced severe diabetes with difficult‐to‐control blood glucose is accompanied by insulin deficiency due to islet β cells loss. Therefore, cellular replacement therapy using insulin‐producing cells is a promising treatment for severe diabetes.

Pancreatic islet transplantation (ITx), a type of cellular replacement therapy, is effective for severe diabetes. The first ITx in Japan was performed in 2004 [1]. Subsequently, the first Japanese prospective multicenter clinical trial was promoted, and efficacy and safety of this therapy were proven [2]. As of 2020, this therapy is covered under the national health care system in Japan. However, the number of cases has not increased dramatically because of limited donors. Therefore, establishing alternative donor sources to human islet cells is a pivotal challenge for promoting this therapy.

Porcine islet xenotransplantation is considered an ideal alternative therapy that resolves the problems associated with islet allotransplantation. Notably, immunity against the xenograft and the risk of zoonoses have limited the feasibility of this therapy [3, 4, 5, 6, 7, 8, 9]. However, the possibility of this therapy has improved with recent progress in gene‐editing technology [10, 11]. Importantly, preclinical studies using nonhuman primates (NHPs) as recipient models are essential for the clinical use of porcine islet xenotransplantation. Accordingly, we performed preclinical porcine islet xenotransplantation using the native Japanese macaque (*Macaca fuscata*), as a NHP member of the macaque family. Japanese macaques are Japanese domestic monkeys that belong to an Old World monkey species, including rhesus and cynomolgus macaques [12]. While the cynomolgus macaque monkey is a representative recipient model for islet xenotransplantation [13, 14], the cost of this monkey increased markedly after the coronavirus disease 2019 (COVID‐19) pandemic. Japanese macaques have been used for various animal studies, including as a model for infection [15]; however, to our knowledge, no previous studies have evaluated porcine islet xenotransplantation using this animal. Therefore, the possibility of the Japanese macaque as a recipient animal model for islet xenotransplantation is undetermined. In particular, there is no information regarding induction of diabetes using this animal.

In this study, we evaluated the suitability of the Japanese macaque as a recipient model for islet xenotransplantation, including identifying the preferred method to induce diabetes.

## 2 Results

### 2.1 Characteristics of the Enrolled Monkeys

Table 1 shows the characteristics of the 20 enrolled monkeys: Model 1: pancreatectomy, *n* = 5; Model 2: pancreatectomy with low‐dose STZ, *n* = 4; Model 3: single‐injection of STZ, *n* = 8; and Model 4: consecutive administrations of low‐dose STZ, *n* = 3. There were no differences among the monkeys in age, body weight, and blood glucose before induction of diabetes.

**TABLE 1:** Characteristics of the enrolled Japanese macaque.

Model	Model 1. Pancreatectomy (*n* = 5)	Model 2. Pancreatectomy with low dose administration of STZ (*n* = 4)	Model 3. Single injection STZ (*n* = 8)	Model 4. Consecutive administration of low dose STZ (*n* = 3)
Age (y)	9.80 ± 0.66	8.25 ± 1.38	8.14 ± 0.94	11.67 ± 3.14
Weight (kg)	13.18 ± 0.57	11.35 ± 1.24	11.4 ± 0.96	8.97 ± 0.38
Blood glucose before induction of diabetes (mg/dL)	64.23 ± 4.49	61.75 ± 4.92	79.67 ± 9.39	72.89 ± 4.71
The dose of STZ (mg/kg)	—	#18004, 19002: 45 #19003: 45, 23, 80 #19004: 70	#19005, 19006, 19007: 100 #19008: 110 #21003: 50, two times #21004, 21005: 50 #23002: 50, 60, 80	#24001: 20 × 5, two times #24002: 20 × 5, 20 × 5, 30 × 5, 30 × 5, 30 × 5, 40 × 5, 50 × 5 #25002: 20 × 5, four times
Procedure of surgery	#18001, 18003, 18005: Total pancreatectomy with reconstruction #18002: Distal pancreatectomy #19001: Subtotal pancreatectomy	#18004: Total pancreatectomy with reconstruction #19002, 19003, 19004: Subtotal pancreatectomy	—	—
Average blood glucose after diabetic induction (mg/dL)	#18001: 149.83 ± 19.91 #18002: 400.00 ± 70.71 #18003: 190.00 ± 49.37 #18005: 168.22 ± 19.65 #19001: 282.00 ± 0.00	#18004: 377.46 ± 57.85 #19002: 63.71 ± 4.24 #19003: 244.31 ± 28.96 #19004: 162.75 ± 12.42	#19005: 202.49 ± 18.25 #19006: 132.73 ± 12.30 #19007: 42.5 ± 16.74 #19008: 111.38 ± 6.24 #21003: 336.49 ± 18.44 #21004: 332.08 ± 50.43 #23002: 70.93 ± 3.45	#24001: 145.91 ± 16.75 #24002: 145.13 ± 14.03 #25002: 126.61 ± 5.33

Abbreviations: y, years; STZ, streptozotocin.

### 2.2 Normal Range of the Blood Glucose Levels for the Japanese Macaque

We measured the blood glucose levels of the 20 enrolled monkeys and three excluded monkeys (#19009, #21002, and #23001) prior to induction of diabetes. There were 139 blood glucose plots. The median normal glucose level was 70.0 mg/dL (range: 31–133 mg/dL), and the mean ± standard deviation (SD) was 70.6 ± 17.1 mg/dL, with a 95% confidence interval (mean ± 1.96 SD) of 37.2–104.0 mg/dL (Figure S1).

### 2.3 Assessment of Achieving Diabetes in Models 1 and 2

As shown in Figure S2 and Table S1, an increase in blood glucose was seen after pancreatectomy in all monkeys in Model 1. The highest blood glucose levels were >200 mg/dL. These data reveal that subtotal pancreatectomy is an effective method of inducing hyperglycemia. However, four of the five monkeys experienced severe adverse events, including adhesion ileus (#18005) and bleeding (#18002). Monkeys #18001 and #18003 developed decreased activities of daily living, including appetite loss and crouching, postoperatively. Therefore, we considered total pancreatectomy an invasive surgery with serious postoperative consequences for the Japanese macaque.

**FIGURE 1:**
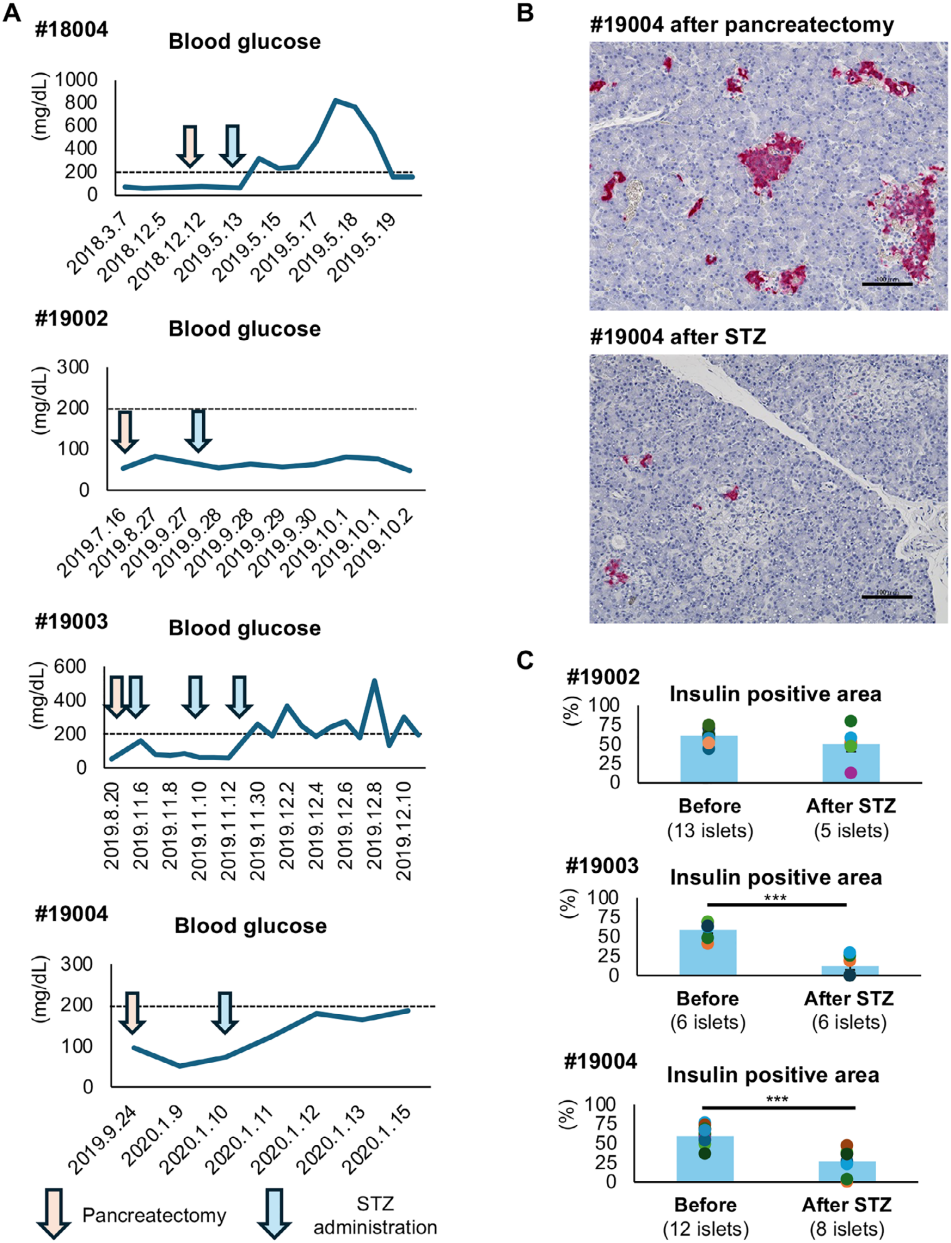
Changes in blood glucose levels before and after induction of diabetes in Model 2. (A) Changes in blood glucose in each monkey (#18004, #19002, #19003, and #19004). The orange arrow indicates the timing of pancreatectomy, and the blue arrow indicates STZ administration. (B) Histologic image of the islets (stained in red) of monkey #19004 before (i.e., after pancreatectomy; left) and after STZ (right). The scale bar indicates 100 µm. (C) The ratio of the insulin‐positive area ( = insulin‐positive area in the islet/total islet area × 100 (%)) before and after STZ in monkeys #19002, #19003, and #19004. The data are presented as mean ± SEM. ***: *p* < 0.001. STZ, streptozotocin; SEM, standard error of the mean.

Four monkeys (#18004 and #19002–#19004) underwent pancreatectomy (1: total pancreatectomy, 3: subtotal pancreatectomy) and STZ (Model 2). While no monkeys developed hyperglycemia after pancreatectomy, two of the four monkeys had blood glucose levels >200 mg/dL after the administration of STZ. Furthermore, significant increases in blood glucose were seen in three monkeys (not in #19002). Specifically, the preoperative blood glucose level of monkey #18004 was 61 and 73 mg/dL. This monkey underwent distal pancreatectomy, and an additional low dose of STZ (45 mg/kg) was administered 5 months after pancreatectomy because hyperglycemia was not achieved (blood glucose: 64 mg/dL). The blood glucose level increased rapidly after STZ administration, and hyperglycemia was maintained thereafter (blood glucose range: 161–823 mg/dL). Regarding monkey #19003, there were no increases in blood glucose for 3 months after subtotal pancreatectomy. This monkey received three additional low‐dose STZ injections. The first dose was 45 mg/kg. The second (23 mg/kg) and third injections (both doses: 80 mg/kg) were performed 4 and 23 days after the first injection, respectively, because hyperglycemia was not achieved with the previous dose(s). The blood glucose level increased immediately to >200 mg/dL 1 day after the third STZ injection and remained high, thereafter (blood glucose range; 133–515 mg/dL). There were no moderate and severe adverse events in any monkey in this model (Figures 1A and S3 and Table S2).

Figure 1B shows the histological findings in the pancreas at pancreatectomy and the end of the assessment after the STZ injections. Pancreatic tissues for histological assessment were obtained from three monkeys (#19002–#19004). A significant decrease in the insulin‐positive area was seen in monkeys #19003 and #19004, while there was no change between pancreatectomy and after STZ in monkey #19002 (Figure 1C). The insulin‐positive area was significantly correlated with the blood glucose level (Figure S4).

Assessment of the results of Model 2 revealed that hyperglycemia was minimally induced after subtotal pancreatectomy without reconstruction. However, additional low‐dose administration of STZ effectively induced diabetes (Figures 1A and S3).

### 2.4 Achieving Diabetes in Model 3: Difficulty Determining the Appropriate Dose of STZ to Induce Diabetes

**FIGURE 2:**
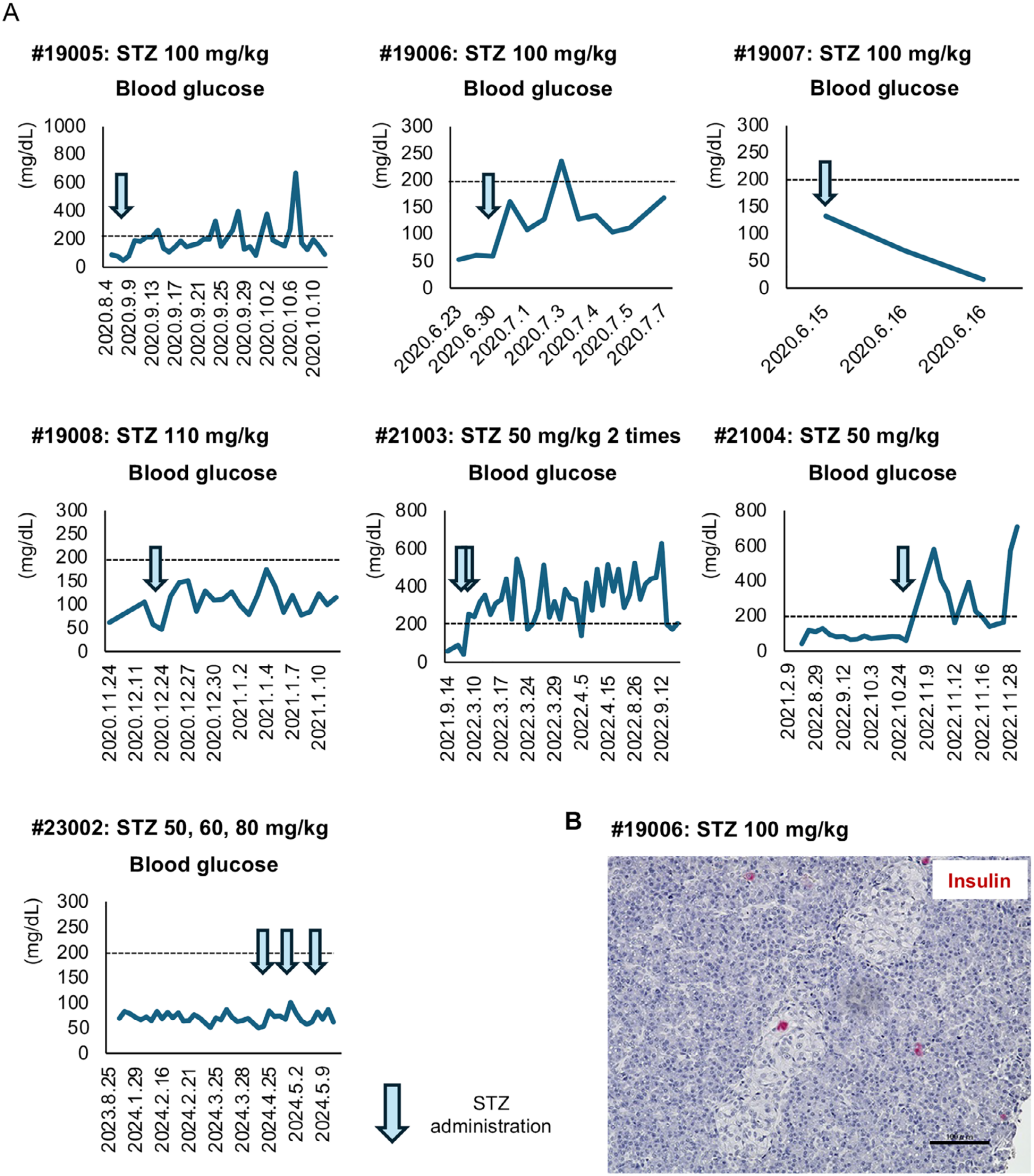
Changes in blood glucose levels before and after induction of diabetes in Model 3. (A) Changes in blood glucose in each monkey (#19005, #19006, #19007, #19008, #21003, #21004, and #23002). The blue arrow indicates the timing of the STZ injections. (B) Histologic image of the islets (stained in red using Warp red) of monkey #19006 after STZ. The scale bar indicates 100 µm. STZ, streptozotocin; SEM, standard error of the mean.

Blood glucose levels in one monkey (#21005) could not be measured after STZ administration because of poor activities of daily living. Therefore, the safety and appropriateness of Model 3 were assessed using the remaining seven monkeys (Table S3). Figure 2A shows the blood glucose levels in the remaining seven monkeys in Model 3. Among them, diabetes (blood glucose >200 mg/dL) was seen in four monkeys (#19005, #19006, #21003, and #21004). Furthermore, a marked increase in blood glucose after STZ administration was seen in five monkeys (#19005, #19006, #19008, #21003, and #21004) (Figure S5). Figure 2B is a histologic image of the pancreas in monkey #19006, which shows that β cells were eliminated by the administration of STZ. This assessment indicated that STZ administration effectively induced diabetes in the Japanese macaque. However, this approach has two limitations. One limitation is difficulty setting the appropriate dose of STZ for the Japanese macaque because the dose of STZ required to achieve diabetes varied between the monkeys. While monkeys #21003 and #21004 developed diabetes after STZ doses of 50 mg/kg, and monkeys #19005 and #19006 developed diabetes after a dose of 100 mg/kg, the blood glucose level in monkey #19008, who received 110 mg/kg of STZ, did not exceed 200 mg/dL. Furthermore, the blood glucose level did not increase in monkey #23002 despite three STZ injections (50, 60, and 80 mg/kg, respectively). These data indicate that the preferable dose of STZ to achieve diabetes depends on the individual Japanese macaque. The other limitation was the frequency of adverse events. STZ‐associated adverse events were seen in all eight monkeys. In particular, moderate and severe adverse events were seen in six of the eight monkeys, as follows: vomiting and anorexia (*n* = 3), liver and kidney disfunction (*n* = 2), hypoglycemia (*n* = 1), and gastrointestinal bleeding (*n* = 1) (Table S3). Figure S6A,B shows the changes in the liver and kidney parameters, namely total bilirubin (T‐Bil), aspartate aminotransferase (AST), alanine aminotransferase (ALT), lactate dehydrogenase (LDH), γ‐glutamyltransferase (γ‐GTP), alkaline phosphatase (ALP), blood urea nitrogen (BUN), and creatinine in monkey #19006, who received 100 mg/kg of STZ and subsequently developed moderate hyperglycemia (Figures 2A and S5). Notably, all liver and kidney values were elevated after STZ administration (Figure S6A,B). Furthermore, extensive fatty liver, renal tubular injury, and atrophic glomeruli were seen in the monkey's tissues, histologically (Figure S6C). These data indicate that it is difficult to induce diabetes in Japanese macaques without adverse events following a single injection of STZ. The dose of STZ required to induce diabetes may be similar to the dose that may induce moderate and severe complications.

### 2.5 Achieving Diabetes in Model 4: A Safer Method to Induce Diabetes

**FIGURE 3:**
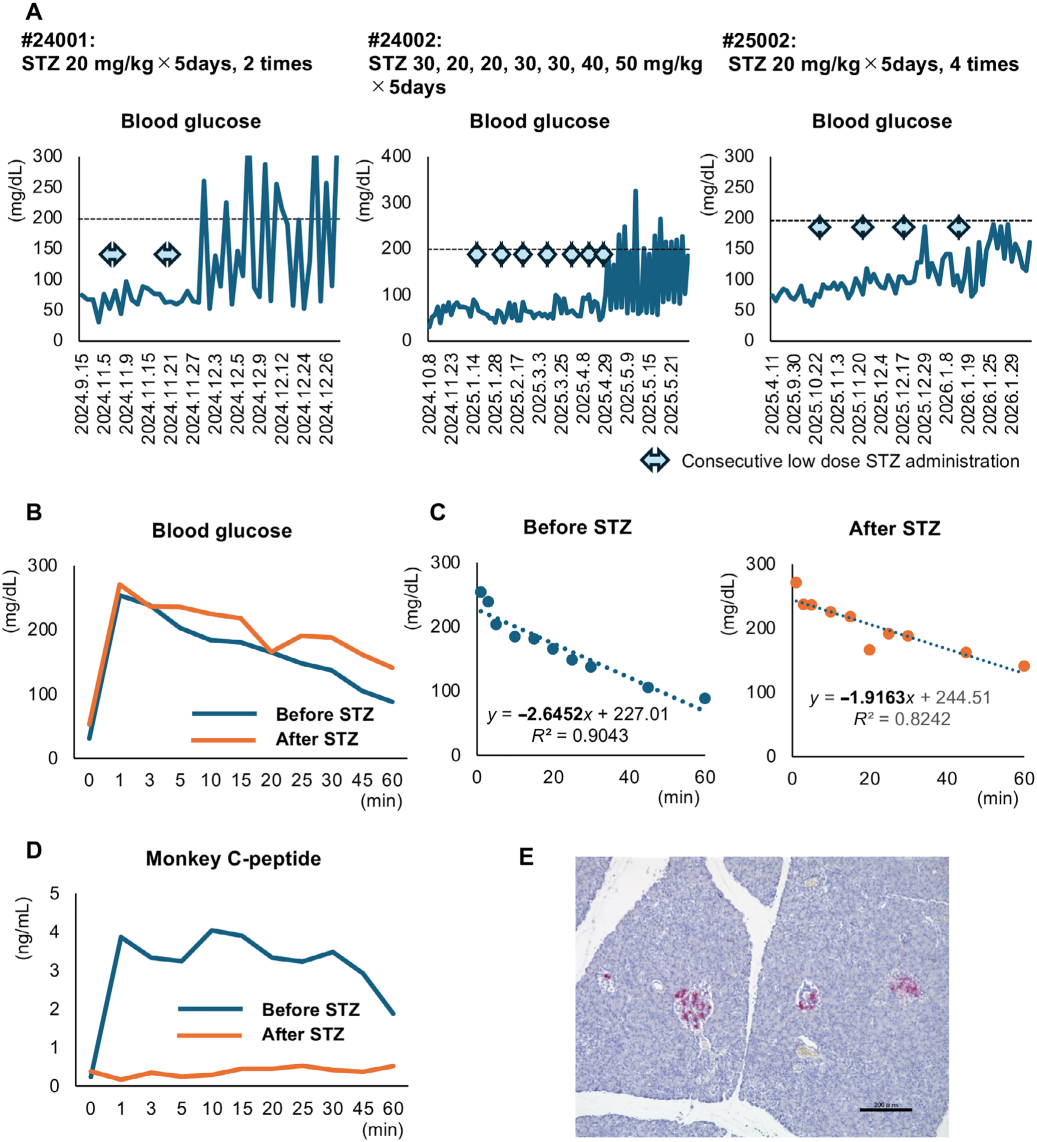
Changes in blood glucose levels before and after induction of diabetes in Model 4. (A) Changes in blood glucose levels in each monkey (#24001, #24002, and #25002). The double‐headed arrows indicate consecutive administrations of low‐dose STZ. (B) Blood glucose changes in diabetic monkey #24001 during the intravenous glucose tolerance test before (blue) and after (orange) STZ administration. (C) Regression analysis between time (*X*‐axis) and blood glucose (*Y*‐axis) after glucose stimulation (left); before STZ administration (blue) (right); and after STZ administration (orange). The outcomes were assessed by a slope of the regression line using all blood glucose levels except those at 0 min. (D) Plasma monkey C‐peptide changes during the intravenous glucose tolerance test. (E) Histologic image of the islets (stained in red) of monkey #21004 after STZ. The original size of the scale bar is 200 µm. STZ, streptozotocin.

**FIGURE 4:**
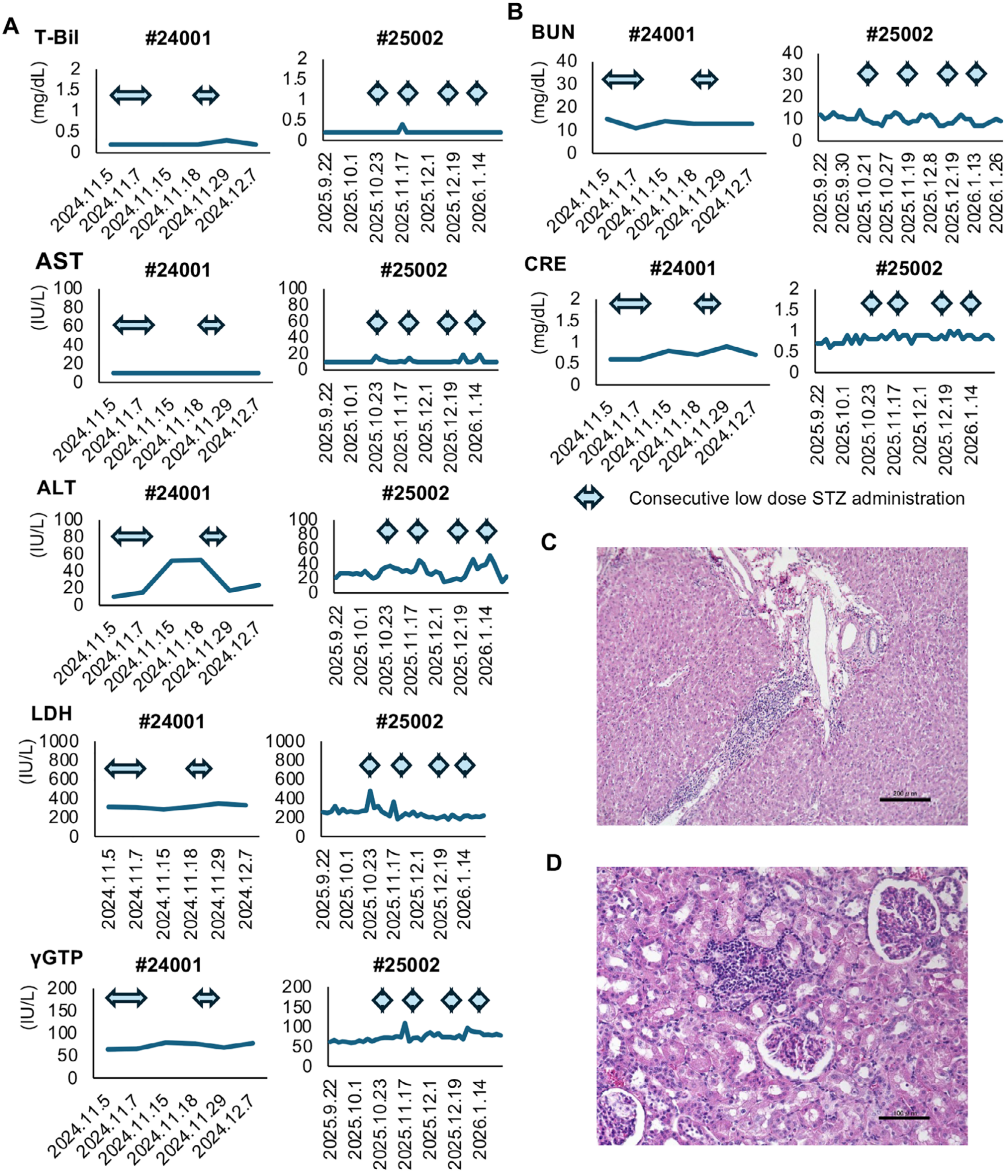
Changes in hepatic and renal functional parameters in monkey #24001 and #25002 before and after STZ administration. (A) The hepatic parameters comprised T‐Bil, AST, ALT, LDH, γ‐GTP, and ALP. (B) The renal parameters comprised BUN and creatinine. The double‐headed arrows indicate the timing of pancreatectomy (left arrowhead) and STZ administration (right arrowhead). (C and D) HE images of the recovered liver (C) and kidney (D) of monkey #24001. The scale bars indicate 200 µm (C) and 100 µm (D). STZ, streptozotocin; T‐Bil, total bilirubin; AST, aspartate aminotransferase; ALT, alanine aminotransferase; LDH, lactate dehydrogenase; γ‐GTP, γ‐glutamyltransferase; ALP, alkaline phosphatase; BUN, blood urea nitrogen; HE, hematoxylin and eosin.

The results in Model 3 confirmed that single‐injection STZ has limited safety at the doses that were used in this model. Therefore, we assessed the consecutive administration of low‐dose STZ as the next step (Model 4). We induced diabetes in three monkeys using this method. Specifically, monkey #24001 received 20 mg/kg of STZ by injection for 5 consecutive days. His blood glucose level increased to >200 mg/dL after the second STZ injection. Monkey #24002 also received consecutive low‐dose injections of STZ and developed hyperglycemia after the injections. However, this monkey was resistant to STZ and required seven consecutive daily injections to achieve diabetes (Figures 3A and S7). Monkey #25002 also received consecutive low‐dose (20 mg/kg) injections of STZ. The maximum blood glucose level in this monkey was 190 mg/dL; however, a significant increase in blood glucose was seen after the low‐dose injections (Figures 3A and S7). Monkey #24001 underwent an intravenous glucose tolerance test (IVGTT). The area under the curve (AUC) and slope of the regression line for blood glucose after glucose administration both increased (i.e., the blood glucose level worsened) after STZ administration (11,100.5 mg/dL × min and −1.92 after STZ vs. 8580 mg/dL × min and −2.65 before STZ; Figure 3B,C). Monkey C‐peptide could not be detected despite glucose administration after STZ, while a response was seen before STZ (Figure 3D). This monkey (#24001) underwent porcine islet xenotransplantation, and the pancreas was recovered 27 days after transplantation because of graft loss. Histological assessment of the pancreas revealed a decrease in the islet‐positive area (Figure 3E). Furthermore, consecutive administrations of low‐dose STZ did not cause moderate and severe adverse events in the liver and kidney in monkeys #24001 and #25002 (Table S4). There were no elevations in the hepatic and renal parameters except for ALT in monkey #24001 (Figure 4A,B). Histologically, fatty change in the liver of monkey #24001 was mild. Glomerular atrophy was seen in this monkey; however, destruction of multiple renal tubules was not seen (Figure 4C,D), similar to the findings in the images in Figure S6C. These data indicate that consecutive administrations of low‐dose STZ safely induced diabetes.

### 2.6 Diabetic Japanese Macaques Are Possible Recipients for Porcine Islet Xenotransplantation

**FIGURE 5:**
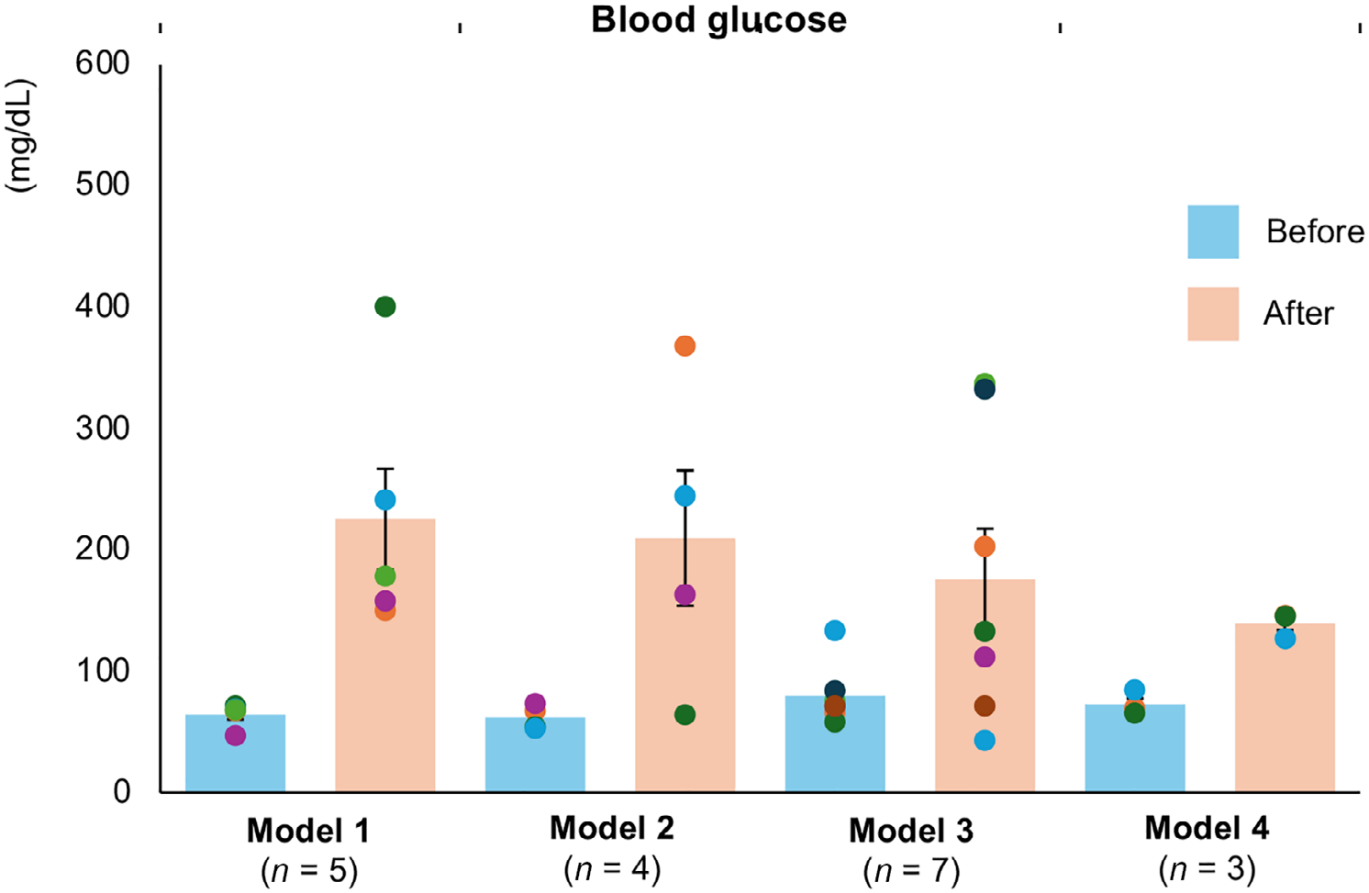
Mean blood glucose levels before and after induction of diabetes in the four models. Mean blood glucose levels before (blue) and after (orange) induction of diabetes in the four models (Model 1: *n* = 5, Model 2: *n* = 4, Model 3: *n* = 7, Model 4: *n* = 3). The data are presented as mean ± SEM. Multiple comparisons were assessed using Tukey's test. SEM, standard error of the mean.

Finally, we assessed the blood glucose changes in the four models. Hyperglycemia was induced in all models, and there was no significant difference in the degree of blood glucose elevation after diabetes induction among the models (225.32 ± 46.49 mg/dL in Model 1, 209.64 ± 64.36 mg/dL in Model 2, 175.51 ± 45.18 mg/dL in Model 3, and 139.22 ± 6.31 mg/dL in Model 4; Figure 5).

**FIGURE 6:**
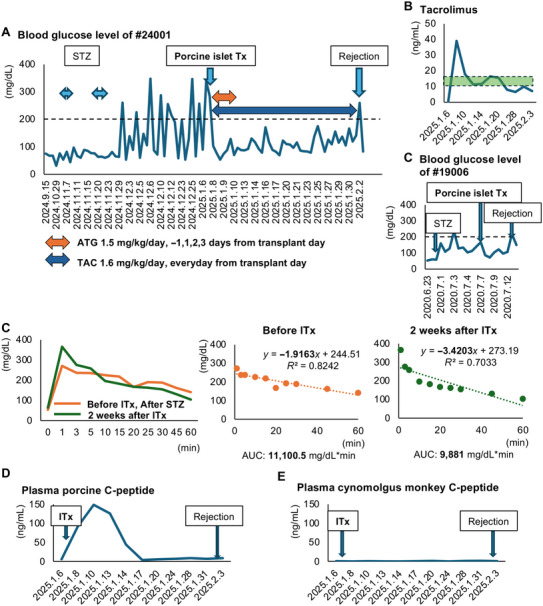
Postoperative course of portal islet xenotransplantation in a diabetic Japanese macaque. (A) Blood glucose levels in monkey #24001 (Model 4) before and after porcine islet xenotransplantation. Immunosuppression using antithymocyte globulin (ATG: 1.5 mg/kg/day, −1, 1, 2, and 3 days from transplant day, as induction) and tacrolimus ((TAC): 1.6 mg/kg/day, from transplant day, as maintenance). (B) Plasma TAC levels before and after porcine islet xenotransplantation. The trough level was 10–15 ng/mL (outlined in green). (C) Blood glucose level of monkey #19006 (Model 3) before and after porcine islet Tx. This monkey did not receive immunosuppressants. (D) Left: Blood glucose changes in diabetic monkey #24001 during the intravenous glucose tolerance test before (orange) and 2 weeks after porcine islet xenotransplantation (green). Right: regression analysis between time (*X*‐axis) and blood glucose (*Y*‐axis) after glucose stimulation: left, before islet transplantation ((ITx): orange) and right: 2 weeks after ITx (green)). The outcomes were assessed by the area under the curve (AUC) values of the blood glucose levels and the slope of the regression line using blood glucose levels except at 0 min. (E and F) Plasma porcine (E) and cynomolgus monkey C‐peptide values (F) of diabetic monkey #24001 after porcine islet xenotransplantation.

We transplanted porcine islets in two diabetic monkeys (i.e., one monkey from Model 3 and the single monkey in Model 4) to assess the therapeutic effects of this treatment. Monkey #24001 was one of the recipients. This monkey was used to establish porcine islet xenotransplantation under the current immunosuppressant protocol for clinical islet (allogeneic) transplantation. The donor pig was an adult male Clawn miniature pig with a body weight of 47 kg (Kagoshima Miniature Swine Research Center, Kagoshima, Japan). The detailed procedure for total pancreatectomy and islet isolation was described in our previous articles [16, 17]. Information on islet isolation and transplanted islets is shown in Table S5. We acquired sufficient numbers of porcine islets for transplantation (40,150 islet equivalents (IEQs)/kg). The functional quality of the islets was acceptable for transplantation (purity: >70%; viability: 85%; and stimulation index, as the ratio of secreted insulin under stimulation with high (16.5 mM), and low glucose concentrations (3.3 mM): 1.49 ± 0.20). Porcine islets were transplanted via the portal vein after laparotomy under general anesthesia using isoflurane (catalog #095–06573; Fujifilm Wako Pure Chemical Co., Osaka, Japan). Regarding immunosuppressants, 1.5 mg/kg/day of antithymocyte globulin (ATG) was used as induction 1 day before, and 1, 2, and 3 days after transplantation, with 1.6 mg/kg/day of tacrolimus (TAC: a calcineurin inhibitor) as maintenance. This regimen imitates the clinical immunosuppressant protocol for ITx in humans [2]. Figure 6A shows the blood glucose levels of monkey #24001 before and after porcine islet xenotransplantation. The blood glucose level decreased immediately and normalized after transplantation. Normoglycemia was maintained for 25 days and increased to >200 mg/dL on Day 26 posttransplantation. We defined this day as the rejection day. Plasma TAC was maintained almost at the trough level throughout the postoperative period (Figure 6B). On the other hand, monkey #19006 who received porcine islet xenotransplantation without immunosuppressants obtained only 6 days normoglycemia (Figure 6C).

The effectiveness of the porcine islet xenotransplantation in #24001 was confirmed in the results of the IVGTT: the AUC and slope of blood glucose improved 2 weeks after ITx: 9881 mg/dL × min and −3.4203 2 weeks after ITx and 11,100.5 mg/dL × min and −1.9163 before ITx (Figure 6D). Notably, plasma porcine C‐peptide was detected up to 11 days after transplantation and was undetectable, thereafter (Figure 6E). Monkey C‐peptide was undetectable throughout the postoperative period (Figure 6F). Laboratory data revealed no elevations in hepatic and renal functional parameters during the postoperative period (Figure S8).

**FIGURE 7:**
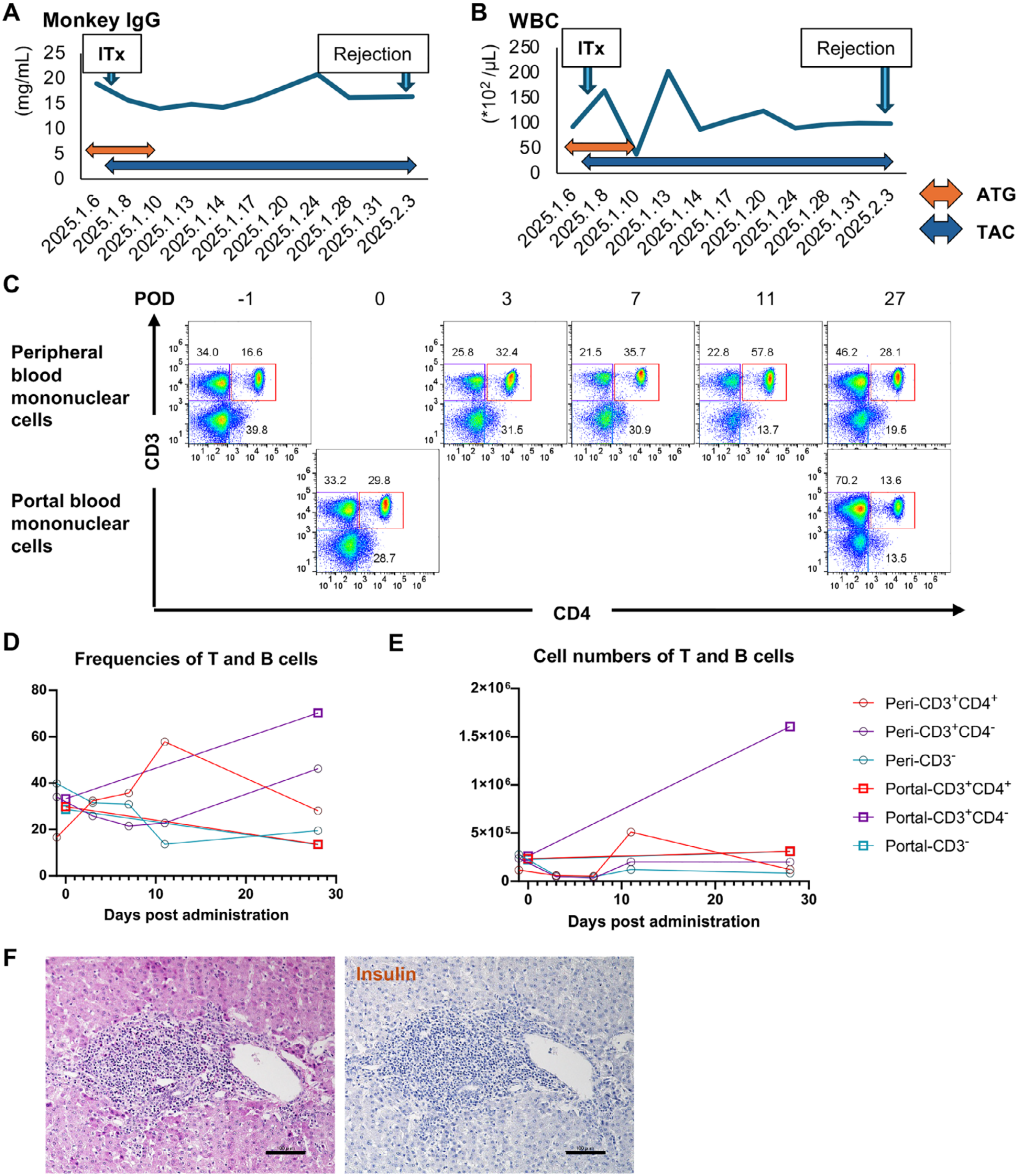
Regulation of immunity under ATG and TAC administration in porcine islet‐xenotransplanted diabetic Japanese macaques. Plasma monkey immunoglobulin G (IgG) levels (A) and white blood cell (WBC) counts (B) of diabetic monkey #24001 after porcine islet xenotransplantation (ITx). (C–E) Flow cytometry analysis using peripheral and portal blood mononuclear cells. (H) Mapping of single‐cell populations for cluster of differentiation (CD)4 (*X*‐axis) and CD3 (*Y*‐axis) before and after porcine islet xenotransplantation. CD3^−^ cells were defined as B cells, CD3^+^CD4^+^ as CD4^+^T cells, and CD3^+^CD4^−^ as CD8^+^T cells. The frequencies (D) and numbers (E) of T and B cells were calculated. (F) Histologic images of the liver of monkey #24001 27 days after transplantation; left: hematoxylin and eosin (HE) image and right: immunostaining for insulin. The scale bar indicates 100 µm. STZ, streptozotocin.

Regarding immunity, monkey immunoglobulin G and white blood cell counts decreased 3 days after transplantation and increased, thereafter (Figure 7A,B). Flow cytometry using peripheral and portal blood mononuclear cells revealed that cell numbers of cluster of differentiation (CD)3^+^CD4^+^ (CD4^+^ T cells), CD3^+^CD4^−^ (CD8^+^ T cells), and CD3^−^ cells (B cells) in peripheral blood mononuclear cells (PBMCs) decreased 3 days posttransplantation and increased 11 days posttransplantation. Regarding portal blood mononuclear cells, the numbers of CD4^+^ T cells, CD8^+^ T cells, and B cells increased 27 days after transplantation compared with those at transplantation (Figure 7C–E).

The liver was recovered from the monkey under general anesthesia using 0.5–2.0% isoflurane 27 days after transplantation. Histological assessment showed infiltration of mononuclear cells around the portal vein and the absence of porcine C‐peptide‐positive regions throughout the liver (Figure 7F). The cellular infiltrations were considered to be destroyed porcine islets attacked by immune‐competent cells. These data reveal that the transplanted porcine islets were successfully engrafted until 11 days after transplantation under the immunosuppression induced by ATG and TAC. However, immunosuppression against the xenograft was only maintained short‐term with this protocol. Subsequent increases in immunoglobulin G, and the numbers of B and T cells eliminated the xenografts, and the islets were completely destroyed by 26 days after transplantation.

In this study, this model (Model 4) is considered the control xenotransplant model and elucidated the limitations of the ATG and TAC antirejection protocol against portal islet xenotransplantation.

## 3 Discussion

Porcine islet xenotransplantation is an ideal alternative treatment for islet allotransplantation. The pig has advantages as an islet donor, one of which is the animal's size. A sufficient number of islets for treating diabetes patients can be acquired from adult porcine islets. The other advantage is the similarity of porcine and human insulin, with a difference of only a single amino acid between these insulins [18]. Therefore, porcine insulin was used historically to treat diabetes before the development of biosynthetic human insulin [19]. Notably, porcine islet xenotransplantation has been considered experimental medicine because of difficulty controlling immunity and the risk of zoonoses [20]. However, improvements in gene‐editing technology have overcome these limitations. For example, porcine islets express major carbohydrate antigens, including galactosyl‐α1,3‐galactose, N‐acetylneuraminic acid, and Sd(a)‐like glycan, which are targets for human antiporcine antibodies and triggers for hyperacute rejection [21]. Regarding this issue, Zhang and colleagues successfully developed galactosyl‐α1,3‐galactose/N‐acetylneuraminic acid/Sda‐like glycan‐negative pigs using CRISPR/Cas9‐mediated gene targeting [22]. Regarding zoonosis, Yang and colleagues reported achieving disruption of porcine endogenous retroviruses (PERV) genes in a porcine cell line, and the authors were able to reduce PERV transmission to another cell line, using CRISPR/Cas9 [23]. Subsequently, trials for developing a suitable donor porcine model for islet xenotransplantation have been promoted and performed. Elliott and colleagues performed immunoisolated neonatal porcine islet xenotransplantation in two patients with type 1 diabetes and confirmed no infection with PERV and short‐term improvement in daily insulin use and hemoglobin A1c levels [24]. Additionally, Matsumoto and colleagues attempted to elucidate the effectiveness and safety of encapsulated neonatal porcine islet xenotransplantation in a Phase 1/2a clinical trial in New Zealand and revealed a reduction in the number of unaware hypoglycemic events in the patients [25].

An NHP animal model is essential to promote preclinical studies of porcine islet xenotransplantation. The cynomolgus macaque is a popular macaque used in various animal studies, including transplantation. For example, Hering and colleagues performed adult porcine islet xenotransplantation in diabetic cynomolgus macaques with immunosuppression using CD25‐ and CD154‐specific monoclonal antibodies, FTY720 or TAC, everolimus, and leflunomide, and reported graft survival of >6 months [26]. However, the price of these macaques has risen dramatically because of the COVID‐19 pandemic. Therefore, there is a need for other NHPs that can be used in preclinical porcine islet xenotransplantation studies. In the present study, we chose the Japanese macaque as a promising islet recipient candidate. This macaque has advantages, such as its size (approximately 10 kg); suitability for various examinations, including drug administration and blood sampling; and reliability as a laboratory animal. This monkey has been used in various medical studies, including in radiation [27] and infectious diseases [15]. In our previous study, we evaluated the possibility of using the Japanese macaque as a recipient model for xenotransplantation [28]. In that study, we administered immunosuppressants, including ATG and TAC, to the monkeys to establish an appropriate protocol to achieve immunosuppression and prevent severe adverse events. We identified the preferred dose of TAC to achieve immunosuppression and prevent severe adverse events, such as multiple organ failure. The preferred TAC dose was markedly higher than the human dose, but similar to that used in cynomolgus macaques [29].

As the next step, we aimed to develop a diabetic animal model using the Japanese macaque. The procedure for diabetes induction in this monkey is not established, with only two previous publications on the topic [30, 31]. We consider the present study as the first to identify the appropriate procedure to induce diabetes and assess the possibility of this monkey as a diabetic recipient for islet xenotransplantation. First, we induced diabetes using only surgery (Model 1). In this assessment, we revealed that surgery, especially total pancreatectomy with reconstruction, effectively induced diabetes in the Japanese macaque. However, moderate and severe complications associated with this method were seen in four of the five monkeys in the model. Therefore, we discarded the procedure as the standard method for inducing diabetes in Japanese macaques. Next, we assessed the possibility of surgery combined with low‐dose STZ. Table S6 shows recent publications describing protocols to induce diabetes using cynomolgus macaques. While many research groups used a single injection of STZ (Model 3 in the present study) to induce diabetes, pancreatectomy combined with low‐dose STZ (Model 2 in the present study) was also widely used (Table S6). In the present study, two of the four monkeys in Model 2 developed diabetes (blood glucose: >200 mg/dL). This model consists of two steps: pancreatectomy and subsequent low‐dose STZ. Subtotal pancreatectomy effectively reduces intrinsic islets and is a safer surgery compared with total pancreatectomy. This is because subtotal pancreatectomy does not require reconstruction and involves a shorter operative time compared with total pancreatectomy. Notable, it may be difficult to induce diabetes using subtotal pancreatectomy because of remnant islets. In our study, no monkeys had elevated blood glucose levels after subtotal pancreatectomy. Additional administration of STZ is a key step in this model. Additionally, the STZ dose can be reduced to induce diabetes because there are fewer intrinsic islets after subtotal pancreatectomy. In our model, elevated blood glucose levels were seen in three of the four monkeys. Furthermore, the lower STZ dose can help prevent STZ‐related adverse events. Indeed, there were no moderate or severe adverse events in the monkeys in Model 2. The limitation of this model is the risk of adhesions caused by laparotomy, which could affect subsequent ITx.

We also assessed the effectiveness of STZ as a major diabetes inducer that has been used widely in various studies, including ITx, diabetic complications [32], and the development of drugs to treat diabetes [33]. The appropriate dose of STZ depends on the species and strain. For example, in mice, a dose of 180 mg/kg of STZ is used for C57BL/6J mice [34] and 220 mg/kg is used for BALB/cAJcl‐nu/nu mice [16]. Compared with mice, rats require a lower dose of STZ: approximately 50 mg/kg is required for both Sprague–Dawley and Wistar rats [35]. Regarding cynomolgus macaques, many studies have indicated that a single intravenous injection of STZ of approximately 100 mg/kg (68–150 mg/kg) is considered appropriate [13, 36, 37, 38, 39, 40, 41, 42, 43, 44, 45, 46, 47, 48, 49, 50, 51, 52, 53, 54]; however, the dose range is wide. Five of the eight monkeys in our Model 3 (single‐injection STZ) developed diabetes; however, the dose of STZ depended on the individual monkey. While the 50 mg/kg STZ dose was sufficient to achieve diabetes in monkeys #21003 and #21004, diabetes could not be induced in monkey #23002 despite the administration of a maximum STZ dose of 80 mg/kg. The limitation of this method is the risk of adverse events, including gastrointestinal, hepatic, and renal impairments. We estimated that the dose of STZ necessary to induce diabetes and result in adverse events was similar. Furthermore, we speculate that the variability in STZ sensitivity is derived from the genetic heterogenicity among the individual. Unlikely to inbred rodent, macaque harbors genetic heterogenicity. We cannot find previous publications about STZ sensitivity in monkey; however, the recent study using mouse revealed that point mutation of gene affected the sensitivity against STZ and diabetes susceptibility [55].

We also assessed consecutive low‐dose STZ injections. We confirmed that diabetes could be induced using this method, and that the method is safer compared with the single‐injection method regarding the prevention of severe adverse events. We also evaluated the usefulness and safety of porcine islet xenotransplantation using this model. We recorded the duration of normoglycemia; islet engraftment; change in immunity under immunosuppressant therapy; and complications associated with ITx, including zoonotic infection and liver and kidney dysfunction due to ITx and immunosuppressants. The results showed that the Japanese macaque with induced diabetes could be used to evaluate the therapeutic effects and safety of porcine islet xenotransplantation.

Importantly, the standard procedure to induce diabetes for Japanese macaques was not established in the present study. We found that surgery has a limitation because of the risk of surgical complications that influence general condition, and difficulty performing subsequent ITx. We also identified that the dose of STZ to induce diabetes in Japanese macaques differed between the individual monkeys. Further studies are necessary to establish this model. However, our results indicate that the Japanese macaque can be used as a recipient model for islet xenotransplantation. Diabetes can be induced by various procedures, including pancreatectomy and STZ administration; however, the risk of adverse events must be considered. Among the possible procedures, consecutive administration of low‐dose STZ might achieve a balance between induction of diabetes and prevention of adverse events. We assessed the preferability of this model by using only three monkeys in this study, and hope that the preferability will be strengthened by further experiences in future.

In the present study, we performed adult porcine islet xenotransplantation in a diabetic Japanese macaque under ATG and TAC administration and confirmed the lack of severe adverse events associated with transplantation and immunosuppression. Graft survival was <1 month; however, we were able to follow the pre‐ and posttransplantation course of porcine islet xenotransplantation using the macaque as a model NHP. As the next step in our research, we will attempt to establish the preferable protocol of immunosuppression by adding mycophenolate mofetil, tumor necrosis factor‐α inhibitors, and other candidates using diabetic Japanese macaques. We will include antiporcine antibody analysis for assessment of engraftment or rejection in the transplant model.

In conclusion, we evaluated the preferable method of inducing diabetes for Japanese macaques. Our results showed that consecutive administration of low‐dose STZ might be preferable to induce diabetes, regarding safety. We also revealed that the Japanese macaque could be used as a recipient model for porcine islet xenotransplantation.

## 4 Materials and Methods

### 4.1 Animals and Ethics

Japanese macaques were provided by the National BioResource Project “Japanese Macaques.” Animals were housed under specific pathogen‐free conditions with free access to food and water. The care of the animals and the experimental procedures complied with the Principles of Laboratory Animal Care (Guide for the Care and Use of Laboratory Animals, 8th edition (National Research Council, 2011)). The experimental protocol was approved by the Animal Care and Use Committee of Fukuoka University (Approval number: 2308040).

### 4.2 Induction of Diabetes Mellitus

#### 4.2.1 Model 1: Pancreatectomy

The aim of model 1 was to induce diabetes in five Japanese macaques (#18001, #18002, #18003, #18005, and #19001) by pancreatectomy (Table 1). Among the five monkeys, three underwent total pancreatectomy with reconstructions of the biliary and gastrointestinal tracts. Subtotal pancreatectomy (>70% pancreatectomy) without reconstruction was performed in the remaining two monkeys (Table S1). Specifically, an abdominal midline incision was made under general anesthesia using 1–1.5% isoflurane. Subtotal pancreatectomy was performed from the pancreatic tail to head with preservation of the spleen. The gastrosplenic ligament and the short gastric vessels were divided, and the pancreatic tail was separated from the spleen while preserving the splenic artery and vein. The pancreas was then mobilized with division of the branches from the splenic vessels. The pancreas was resected at its head, right side of the portal vein, and as close to the duodenum as possible. Blood flow to the duodenum was preserved. The main pancreatic duct was dissected using 4‐0 Vicryl suture (Ethicon Inc., Somerville, NJ, USA). The common bile duct was preserved (Figure S9).

#### 4.2.2 Model 2: Pancreatectomy, With Low‐Dose STZ Injections

Subsequent to Model 1, in Model 2, we performed pancreatectomy with low‐dose STZ injections in four monkeys (#18004, #19002, #19003, and #19004). Three monkeys (#19002–#19004) underwent subtotal pancreatectomy, and monkey #18004 underwent total pancreatectomy. The surgical procedures were as described, above. The dose of STZ (Zanosar, Nobelpharma Co., Ltd., Tokyo, Japan) was 45 mg/kg in three monkeys (#18004, #19002, and #19003) and 70 mg/kg in monkey #19004. Monkey #19003 received three STZ injections (45, 23, and 80 mg/kg, respectively) because hyperglycemia was not achieved with the first two doses (Table S2).

#### 4.2.3 Model 3: Single‐Injection of STZ

In Model 3, we attempted to induce diabetes in eight monkeys (#19005–#19008, #21003–#21005, and #23002) with a single injection of intravenous STZ. The individual doses are presented in Table S3. Briefly, 50 mg/kg of STZ was administered to four monkeys (#21003–#21005 and #23002). One monkey among these four (#23002) received additional injections (60 and 80 mg/kg, respectively) because hyperglycemia was not achieved with the first doses. The remaining four monkeys received higher doses of STZ (100 mg/kg for three: #19005–#19007 and 110 mg/kg for one: #19008). STZ was injected via a central venous access port into the inferior vena cava. After the injection, 500 mL of acetated Ringer's solution containing 5% glucose was administered by intravenous drip to prevent dehydration and renal injury, which are adverse events associated with STZ. If hyperglycemia was not achieved, readministration of STZ was discussed. Multiple STZ injections were administered at least 2 weeks apart.

#### 4.2.4 Model 4: Consecutive Administration of Low‐Dose STZ

In Model 4, we attempted to induce diabetes using consecutive (daily for 5 days) low‐dose STZ injections in three monkeys (#24001, #24002, and #25002). The schedules and doses are shown in Table S4.

### 4.3 Definition of the Achievement of Diabetes Mellitus

Nonfasting blood glucose levels were measured to detect diabetes (blood glucose >200 mg/dL) using the Glutest Mint kit (Sanwa Kagaku Kenkyusho Co., Ltd., Nagoya, Japan), in accordance with the manufacturer's instructions. The definition of the diabetes is based on some previous publications [40, 53, 54].

### 4.4 Insertion of the Central Venous Access Port

A central venous access port was inserted prior to STZ administration. A 6‐Fr central venous access port (Bard X‐Port isp, #0607530; Becton, Dickinson and Company, Franklin Lakes, NJ, USA) was inserted via the femoral vein, and the tip of the tube was fixed in the inferior vena cava.

### 4.5 Monitoring Blood Glucose and Plasma C‐Peptide

The achievement of diabetes was assessed by monitoring blood glucose levels. Blood samples were collected from the central venous access port and measured using the Glutest Mint kit (Sanwa Kagaku Kenkyusho Co., Ltd.), as stated. Regarding plasma C‐peptide, 1 mL blood samples were heparinized using <2 µL of heparin. Plasma was extracted from the samples by centrifugation at 840 *g* rpm for 15 min. The C‐peptide level in the plasma was measured using an enzyme‐linked immunosorbent assay (ELISA) kit (Cynomolgus Monkey C‐Peptide ELISA Kit, NBP2‐59957; Novus Biological, Centennial, CO, USA), in accordance with the manufacturer's instructions. The absorbance at 450 nm (optical density 450) was determined using an iMark Microplate Absorbance Reader with Microplate Manager Software 6 (Bio‐Rad, Hercules, CA, USA).

### 4.6 IVGTT  

The monkeys were fasted overnight (15 h) prior to the IVGTT, which was performed under general anesthesia. An intravenous catheter (18 G) was inserted into the antebrachial vein for blood sample collection before glucose stimulation. Next, 25% glucose solution (0.5 g/kg body weight) was injected into the inferior vena cava via the central venous access port. Blood glucose levels were measured at 0, 1, 3, 5, 10, 15, 20, 25, 30, 45, and 60 min after glucose injection. The outcomes were assessed by the AUC of the blood glucose levels and the slope of the regression line using all blood glucose values except those at 0 min. The plasma samples used to detect C‐peptide were collected when samples were collected for blood glucose measurement.

### 4.7 Laboratory Parameters

The following were measured using the Celltac α analyzer (Nihon Kohden Europe GmbH, Rosbach, Germany): white blood cell count, hemoglobin level, and platelet count. Plasma samples for biochemical examination were isolated from heparinized blood by centrifugation at 2,000 *g* for 5 min. Liver function parameters (T‐Bil, AST, ALT, LDH, γ‐GTP, and ALP), kidney function parameters (BUN and creatinine), and nutritional status indicators (total protein and albumin) were measured using the Spotchem EZ SP4430 (ARKRAY, Inc., Kyoto, Japan). Plasma TAC levels were measured by SRL, Inc. (Tokyo, Japan).

### 4.8 Isolation of PBMCs

PBMCs were isolated from blood samples using BD Vacutainer CPT Mononuclear Cell Preparation Tubes—Sodium Heparin (catalog #362753; Becton, Dickinson and Company), in accordance with the manufacturer's instructions. Portal blood mononuclear cells were collected using the same method.

### 4.9 Flow Cytometry

Flow cytometry using PBMCs from Monkey #24001 was performed 1 day before, and 3, 7, 11, and 27 days after porcine islet xenotransplantation during immunosuppression (thymoglobulin: −1, 1, 2, and 3 days after transplantation; TAC: 0–25 days after transplantation). Briefly, PBMCs were labeled by incubation using Alexa Fluor 488 mouse anti‐human CD3 [SP34‐2] (catalog #557705; Becton, Dickinson and Company), PE mouse immunoglobulin G 2b, κ Isotype Control Antibody (MPC‐11) (catalog #400311; BioLegend, San Diego, CA, USA), and allophycocyanin mouse anti‐human CD4 (L200) (catalog #551980; Becton, Dickinson and Company). Flow cytometry was performed using the BD Accuri C6 Plus flow cytometer (Becton, Dickinson and Company). Dead cells were gated using 7‐amino‐actinomycin D (Becton, Dickinson and Company), and lymphocytes were identified by forward and side scatter. Doublets, determined by forward scatter height and width, were excluded from the analysis. Singlets were then gated as CD3^−^ (B cells), CD3^+^CD4^+^ (CD4^+^ T cells), and CD3^+^CD4^−^ (CD8^+^ T cells).

### 4.10 Histological Assessment

The liver, one kidney, and the pancreas were recovered from the two monkeys (one from Model 3 and the single monkey from Model 4) who underwent xenotransplantation, under general anesthesia using 0.5–2.0% isoflurane. After recovery, the monkeys were euthanized with an intravenous injection of thiopental sodium (200 mg/kg). The recovered organs were fixed using 10% formalin. Three‐micrometer‐thick sections were either stained with hematoxylin and eosin or underwent immunohistochemistry (for insulin to identify islets, and for porcine C‐peptide to identify porcine islets). The primary antibodies used in the analysis were guinea pig anti‐insulin (catalog #A056401‐2, RRID:AB_2617169; 1:100; Agilent, Dako, Tokyo, Japan) and mouse anti‐pig C‐peptide (catalog #MAA447Po21; 1:200; Cloud‐Clone Corp., Katy, TX, USA). After incubation with a primary antibody, donkey anti‐mouse immunoglobulin G (H+L) Alexa488 (catalog #715‐547‐003, RRID:AB_2340851; 1:100; Jackson ImmunoResearch Laboratories, Inc., West Grove, PA, USA) was used as a secondary antibody. The Warp Red Chromogen Kit (Biocare Medical LLC, Depew, NY, USA) was used for staining. Histological images were obtained using a BZ‐X700 microscope (Keyence, Itasca, IL, USA), and immunostaining was quantified using ImageJ software (https://imagej.nih.gov/ij/index.html; National Institutes of Health, Bethesda, MD, USA).

### 4.11 Porcine Pancreatic Islet Isolation

Detailed procedures for porcine pancreatic islet isolation were described in our previous article [17]. Briefly, a collagenase solution containing a mammalian tissue‐free version of Liberase (Liberase MTF; 0.5 g per 1 vial; Roche Diagnostics GmbH, Mannheim, Germany (0.5 g per vial) and thermolysin (15 mg per vial) (catalog #05339880001; Roche CustomBiotech, Penzberg, Germany) was instilled into the pancreas via a catheter placed in the pancreatic duct. The distended pancreas was resected and placed in a Ricordi chamber in semi‐closed circuits within a water bath. The pancreas was digested by the shaking of the Ricordi chamber and the circulation of warmed collagenase solution. The digested tissue was diluted in RPMI 1640 solution (catalog #11875085; Gibco; Thermo Fisher Scientific, Inc., Waltham, MA, USA) containing 10% inactivated fetal bovine serum (catalog #26140079; Gibco; Thermo Fisher Scientific, Inc.) and then collected in Belzer UW cold storage solution (Bridge to Life, London, UK), which included ulinastatin. The purification process was performed using an IBM/COBE 2991 cell processor (Terumo BCT, Tokyo, Japan) by centrifugation with a continuous density gradient between 1.077 and 1.100 g/cm^3^ created using Optiprep (catalog #ST‐07820; Veritas Corp., Tokyo, Japan). After centrifugation, gradient density solutions containing highly‐purified islets (≥70%) were collected.

### 4.12 Assessing the Quality of the Porcine Islets

The purity, viability, and glucose‐stimulated insulin secretion levels of the islets were assessed in accordance with our protocol [16]. Briefly, purity was determined using dithizone (catalog #D5130; Sigma–Aldrich, St. Louis, MO, USA) staining. Islet viability was evaluated using Hoechst 33342 nucleic acid stain (catalog #H1399; Invitrogen, Waltham, MA, USA) and propidium iodide (catalog #P1304MP; Invitrogen) double staining. Glucose‐stimulated insulin secretion was evaluated by measuring insulin secreted from porcine islets incubated with 3.3 mM (low) and 16.5 mM (high) glucose solutions. Porcine insulin was measured using an ELISA kit (LBIS Porcine Insulin ELISA Kit; catalog #AKRIN‐013T; Fujifilm Wako Shibayagi Corp., Shibukawa, Japan). The stimulation index, as the ratio of the insulin concentrations generated under high‐ and low‐glucose stimulation, was then calculated.

### 4.13 Porcine Islet Xenotransplantation

The porcine islets were collected in 20 mL of CMRL1066 solution (CMRL1066‐Custom01; Gmep, Kurume, Japan), including a small amount of heparin. The recipient monkey (in both cases) underwent laparotomy under general anesthesia using 0.5–2.0% isoflurane. The duodenum was mobilized, and the trunk of the portal vein was identified behind the pancreas. A 20‐G intravenous catheter (Becton, Dickinson and Company) was canulated into the trunk, and the porcine islets were immediately injected via the catheter. A TachoSil tissue sealing sheet (CSL Behring K.K., Tokyo, Japan) was used for hemostasis.

### 4.14 Statistical Analysis

Blood glucose and plasma C‐peptide values, and the AUCs and slopes of the regression lines in the IVGTTs were compared among the models. Multiple comparisons were assessed using Dunnett's test and Tukey's test. Data are presented as the mean ± SD or standard error of the mean. Significant differences were defined as *p* < 0.05, and all tests were two‐sided. Statistical analyses were performed using JMP version 18.0.0 (SAS Institute Inc., Cary, NC, USA).

## Author Contributions

NS planned the study, performed all the experiments and data analyses except the histological assessment, and wrote the first draft of the manuscript. GY performed the porcine islet isolation and revised the manuscript. RK performed the porcine islet isolation and histological and flow cytometry assessments and revised the draft of the manuscript. ST performed anesthesia and revised the manuscript. SK supported the study design and revised the draft of the manuscript. NS and SK are the guarantors of the work, and as such, take responsibility for the contents of the article. All authors have read and approved the final manuscript.

## Funding

The present study was funded by an intramural grant from Fukuoka University (#GR2420, to NS), the Japan Diabetes Foundation and Sanofi K.K. (#240434 to NS), Takahashi Industrial and Economic Research Foundation (#250038 to NS), and a Grant‐in‐Aid for Scientific Research “KAKENHI” (#25K11992, to NS, and #24K02514, to SK).

## Conflicts of Interest

The authors declare no conflicts of interest.

## Ethics Statement

Japanese macaques were provided by the National BioResource Project “Japanese Macaques.” Animals were housed under specific pathogen‐free conditions with free access to food and water. The care of the animals and the experimental procedures complied with the Principles of Laboratory Animal Care (Guide for the Care and Use of Laboratory Animals, 8th edition (National Research Council, 2011)). The experimental protocol was approved by the Animal Care and Use Committee of Fukuoka University (Approval number: 2308040). This study was reported in accordance with the ARRIVE guidelines. All experiments were performed in accordance with relevant guidelines and regulations.

## Supporting information



Supporting File: 1

## Data Availability

The datasets used and/or analyzed in the present study are available from the corresponding author upon reasonable request.
