# Supplementary material for: The Japanese Macaque as a Diabetes Recipient Animal Model for Porcine Islet Xenotransplantation
Source: MedComm (2020). 2026 Apr 8;7(4):e70726. doi: 10.1002/mco2.70726 (PMC13062647; doi:10.1002/mco2.70726)
Supplement: Supplementary file 1 — Supporting File: 1 [file MCO2-7-e70726-s001.pdf]

The Japanese Macaque as a Diabetes Recipient Animal Model for Porcine Islet Xenotransplantation

Short running title: Japanese Macaque for Islet XenoTx

Naoaki Sakata, MD[1,2,*]; Gumpei Yoshimatsu, MD[1,2]; Ryo Kawakami, MS[1,2]; Seiichi Tanaka, DVM, DJCLAM[3], Shohta Kodama, MD[1,2]

[1]Department of Regenerative Medicine and Transplantation, Faculty of Medicine, Fukuoka University, 7-45-1 Nanakuma, Jonan-ku, Fukuoka 814-0180, Japan

[2]Center for Regenerative Medicine, Fukuoka University Hospital, 7-45-1 Nanakuma, Jonan-ku, Fukuoka 814-0180, Japan

[3]Center for Experimental Animals, Fukuoka University, 7-45-1 Nanakuma, Jonan-ku, Fukuoka 814-0180, Japan

***Corresponding author:** Naoaki Sakata, MD

Department of Regenerative Medicine and Transplantation

Faculty of Medicine, Fukuoka University

7-45-1 Nanakuma, Jonan-ku, Fukuoka 814-0180, Japan

Tel: +81-92-801-1011 (ext. 3631)

Fax: +81-92-801-1019

E-mail: naoakisakata@fukuoka-u.ac.jp

ORCID ID: https://orcid.org/0000-0003-3522-3995

E-mail addresses: NS: naoakisakata@fukuoka-u.ac.jp, GY: gyoshimatsu@fukuoka-

u.ac.jp, RK: ryok@fukuoka-u.ac.jp, ST: denchu@fukuoka-u.ac.jp, SK: shohtakodama@gmail.com

Table S1. Characteristics of the Japanese macaques in Model 1 (pancreatectomy)

Monkey ID	#18001	#18002	#18003	#18005	#19001
Age (years)	10	10	9	8	12
Weight (kg)	14.2	14.3	13.4	11.2	12.8
Protocol	Total pancreatectomy	Distal pancreatectomy (70%)	Total pancreatectomy	Total pancreatectomy	Subtotal pancreatectomy
Blood glucose before DM induction (mg/dL)	56 – 74	56 – 87	55 – 79	45, 48	52, 84
Blood glucose after DM induction (mg/dL)	92 – 221, $p = 0.01$	300, 500, $p = 0.19$	176 – 304, $p = 0.04$	46 – 237, $p = 0.001$	75, 282, $p = 0.48$
Achievement of DM (>200mg/dL)	Yes	Yes	Yes	Yes	Yes
Follow up	7 days	2 hours	2 days	11 days	147 days
Adverse events	Surgical stress	Bleeding	Surgical stress	Adhesion ileus	None

ID, identification; DM, diabetes mellitus

Table S2. Characteristics of the monkeys in Model 2 (pancreatectomy with low-dose STZ)

Monkey ID	#18004	#19002	#19003	#19004
Age (years)	10	11	7	5
Weight (kg)	15.0	10.0	10.8	9.6
Protocol	Total pancreatectomy with 45mg/kg STZ	Subtotal pancreatectomy with 45mg/kg STZ	Subtotal pancreatectomy with 45, 23, 80 mg/kg STZ	Subtotal pancreatectomy with 70mg/kg STZ
Blood glucose before DM induction (mg/dL)	61, 73	54	53	50, 96

**Blood glucose after DM induction (mg/dL)**	105 – 400, $p < 0.0001$	44 – 83	60 – 365	51 – 186, $p = 0.07$
**Achievement of DM (>200mg/dL)**	Yes	No	Yes	No
**Follow up**	23 days	46 days	121 days	119 days
**Adverse events**	None	None	None	None

ID, identification; STZ, streptozotocin; DM, diabetes mellitus

Table S3. Characteristics of the monkeys in Model 3 (single-injection of STZ)

Monkey ID	#19005	#19006	#19007	#19008
**Age (years)**	9	8	8	11
**Weight (kg)**	14.8	12.4	14.4	9.8
**Protocol**	STZ 100 mg/kg	STZ 100 mg/kg	STZ 100 mg/kg	STZ 110 mg/kg
**Blood glucose before DM induction (mg/dL)**	46 – 90	53 – 61	133	58 – 105
**Blood glucose after DM induction (mg/dL)**	77 – 670, $p < 0.0001$	86 – 236, $p <0.001$	16, 69	47 – 175, $p = 0.03$
**Achievement of DM (>200mg/dL)**	Yes	Yes	No	No
**Follow up**	59 days	14 days	1 days	46 days
**Adverse events**	Vomiting	Vomiting, Anorexia, Liver and kidney dysfunction	Hypoglycemia	Vomiting, Anorexia

Monkey ID	#21003	#21004	#21005	#23002
Age (years)	5	5	5	11
Weight (kg)	8.0	9.9	11.9	10.5
Protocol	STZ 50 mg/kg, 2 times	STZ 50 mg/kg	STZ 50 mg/kg	STZ 50, 60, 80 mg/kg
Blood glucose before DM induction (mg/dL)	58, 92	43 – 130	51 – 92	51 – 87
Blood glucose after DM induction (mg/dL)	42 – 628, $p < 0.001$	140 – 709, $p < 0.001$	No records	50 – 101, $p = 0.98$
Achievement of DM (>200mg/dL)	Yes	Yes	No	No
Follow up	205 days	29 days	5 days	29 days
Adverse events	Vomiting	Vomiting, Anorexia, Dehydration	Gastrointestinal bleeding	Liver and kidney dysfunction

ID, identification; STZ, streptozotocin; DM, diabetes mellitus

Table S4. Characteristics of the monkeys in Model 4 (consecutive administration of low-dose streptozotocin)

Monkey ID	#24001	#24002	#25002
Age (years)	4	16	15
Weight (kg)	8.2	8.9	9.8
Protocol	STZ 20 mg/kg 5 days 2 times	STZ 20 mg/kg 5 days 2 times, 30	STZ 20 mg/kg 5 days 4 times

		mg/kg 5 days 3 times, 40 mg/kg 5 days 1 time, 50 mg/kg 5 days 1 time	
**Blood glucose before DM induction (mg/dL)**	31 – 75	31 – 84	58 - 118
**Blood glucose after DM induction (mg/dL)**	53 – 348, p < 0.001	59 – 326, p <0.001	78 – 190 p <0.001
**Achievement of DM (>200mg/dL)**	Yes	Yes	No
**Follow up**	49 days	28 days	107 days
**Adverse events**	Appetite loss	None	Vomiting

ID, identification; STZ, streptozotocin; DM, diabetes mellitus

Table S5. Information on porcine islet isolation and transplanted islets

**Pancreas weight (g)**	64
**Warm ischemic time (min)**	2
**Cold ischemic time (min)**	132
**Collagenase**	Liberase MTF/Thermolysin (Roche, Basel, Switzerland)
**Purification**	Using COBE2991 (Terumo BCT, Tokyo, Japan)
**Islet yields for transplantation (IEQs)**	309,155 (40,150 IEQs/kg)
**Purity (%)**	>70
**Viability (%)**	85

**Stimulation index in glucose stimulated insulin secretion**	1.49 ± 0.20

MTF, mammalian tissue-free; IEQs, islet equivalents

Table S6. Published protocols on the induction of diabetes for islet transplantation using the cynomolgus macaque

Author, year and reference	Group	Method	Model classification	Definition of Diabetes
Leishman DJ, 2024[36]; Graham ML, 2022[13]	Minnesota	80-100 mg/kg of streptozotocin, Zanosar, single IV injection	Model 3	BG: >300 mg/dL, C-peptide: <0.3 ng/mL
Ellis GI, 2022[37]	Pennsylvania	85 mg/kg of streptozotocin, Zanosar, single IV injection	Model 3	BG: >250 mg/dL, C-peptide: <0.5 ng/mL
Kenyon N, 2021[38], Berman DM, 2016[39]	Miami	100 mg/kg of streptozotocin, Zanosar, single IV injection	Model 3	C-peptide: <0.3 ng/mL
Kim GS, 2021[46], Kim GS, 2020[47], Oh BJ, 2018[48], Park H, 2017[49]	Sungkyunkwan	60-80 mg/kg of streptozotocin, single IV injection with subtotal pancreatectomy (>70%)	Model 2	BG: >250 mg/dL, C-peptide: <0.5 ng/mL
Liu Z, 2020[40]	Shenzhen	100 mg/kg of streptozotocin, IV	Model 3	BG: >200 mg/dL, C-peptide: <0.5 ng/mL
Kim JM, 2019[50]	[1]Seoul	110 mg/kg of streptozotocin, single IV injection or 60 mg/kg of streptozotocin, single IV injection with partial pancreatectomy	Model 2 and 3	BG: >250 mg/dL
Oura T, 2019[41,42]	Harvard	75 mg/kg of streptozotocin, Zanosar, single IV injection	Model 3	BG: >250 mg/dL, C-peptide: <0.05 ng/mL
Bottino R, 2014[43], Nagaraju S,	Pittsburgh	125-150 mg/kg of streptozotocin, Zanosar, single IV injection	Model 3	

2014[44]				
Koulmanda M, et al. 2014[54], 2012[53]	Harvard	55 mg/kg of streptozotocin, Zanosar, single IV injection with partial pancreatectomy (70-80%)	Model 2	BG: >200 mg/dL
Watanabe M, 2013[51], Yoshida T, 2012[52]	Hokkaido	Total pancreatectomy without duodenectomy or splenectomy	Model 1	Maintain 100 – 200 mg/dL in BG[2]; C-peptide: <0.3 ng/mL[3]
Zhou H, 2012[45]	Pittsburgh	150 mg/kg of streptozotocin, Zanosar, single IV injection	Model 3	C-peptide: <0.1 ng/mL

BG, blood glucose; IV, intravenous

13. Graham ML, Ramachandran S, Singh A, et al. Clinically available immunosuppression averts rejection but not systemic inflammation after porcine islet xenotransplant in cynomolgus macaques. Am J Transplant. 2022;22(3): 745–760.

36. Leishman DJ, Oppler SH, Stone LLH, et al. Targeted mapping and utilization of the perihepatic surface for therapeutic beta cell replacement and retrieval in diabetic non-human primates. Front Transplant. 2024;3: 1352777.

37. Ellis GI, Coker KE, Winn DW, et al. Trafficking and persistence of alloantigen-specific chimeric antigen receptor regulatory T cells in Cynomolgus macaque. Cell Rep Med. 2022;3(5): 100614.

38. Kenyon NS, Willman MA, Han D, et al. Extended survival versus accelerated rejection of nonhuman primate islet allografts: Effect of mesenchymal stem cell source and timing. Am J Transplant. 2021;21(11): 3524–3537.

39. Berman DM, Molano RD, Fotino C, et al. Bioengineering the Endocrine Pancreas: Intraomental Islet Transplantation Within a Biologic Resorbable Scaffold. Diabetes. 2016;65(5): 1350–1361.

40. Liu Z, Lu Y, Hu W, et al. Induction of diabetes in cynomolgus monkey with one shot of analytical grade streptozotocin. Animal Model Exp Med. 2020;3(1): 79–86.

41. Oura T, Hotta K, Lei J, et al. Immunosuppression With CD40 Costimulatory Blockade Plus Rapamycin for Simultaneous Islet-Kidney Transplantation in Nonhuman Primates. Am J Transplant. 2017;17(3): 646–656.

42. Oura T, Hotta K, Rosales I, et al. Addition of Anti-CD40 Monoclonal Antibody to Nonmyeloablative Conditioning With Belatacept Abrogated Allograft Tolerance Despite Induction of Mixed Chimerism. Transplantation. 2019;103(1): 168–176.

43. Bottino R, Wijkstrom M, van der Windt DJ, et al. Pig-to-monkey islet xenotransplantation using multi-transgenic pigs. Am J Transplant. 2014;14(10): 2275–2287.

44. Nagaraju S, Bertera S, Funair A, et al. Streptozotocin-associated lymphopenia in cynomolgus monkeys. Islets. 2014;6(3): e944441.

45. Zhou H, van der Windt DJ, Dons EM, et al. A syndrome of severe hypoglycemia and acidosis in young immunosuppressed diabetic monkeys and pigs-association with sepsis. Transplantation. 2012;94(12): 1187–1191.

46. Kim GS, Cho CW, Lee JH, et al. Optimal allogeneic islet dose for transplantation in insulin-dependent diabetic Macaca fascicularis monkeys. Sci Rep. 2021;11(1): 8617.

47. Kim GS, Lee JH, Shin DY, et al. Integrated whole liver histologic analysis of the allogeneic islet distribution and characteristics in a nonhuman primate model. Sci Rep. 2020;10(1): 793.

48. Oh BJ, Jin SM, Hwang Y, et al. Highly Angiogenic, Nonthrombogenic Bone Marrow Mononuclear Cell-Derived Spheroids in Intraportal Islet Transplantation. Diabetes. 2018;67(3): 473–485.

49. Park H, Park JB, Kim JH, et al. Simultaneous Subtotal Pancreatectomy and Streptozotocin Injection for Diabetes Modeling in Cynomolgus Monkeys. Transplant Proc. 2017;49(5): 1142–1149.

50. Kim JM, Shin JS, Min BH, et al. JAK3 inhibitor-based immunosuppression in allogeneic islet transplantation in cynomolgus monkeys. Islets. 2019;11(5): 119–128.

51. Watanabe M, Yamashita K, Suzuki T, et al. ASKP1240, a fully human anti-CD40 monoclonal antibody, prolongs pancreatic islet allograft survival in nonhuman primates. Am J Transplant. 2013;13(8): 1976–1988.

52. Yoshida T, Suzuki T, Watanabe M, et al. Induction of insulin-dependent diabetes mellitus by total pancreatectomy for pancreatic islet transplantation in cynomolgus monkeys. J Hepatobiliary Pancreat Sci. 2012;19(6): 661–666.

53. Koulmanda M, Qipo A, Fan Z, et al. Prolonged survival of allogeneic islets in cynomolgus monkeys after short-term triple therapy. Am J Transplant. 2012;12(5): 1296–1302.

54. Koulmanda M, Sampathkumar RS, Bhasin M, et al. Prevention of nonimmunologic loss of transplanted islets in monkeys. Am J Transplant. 2014;14(7): 1543–1551.

Figure S1

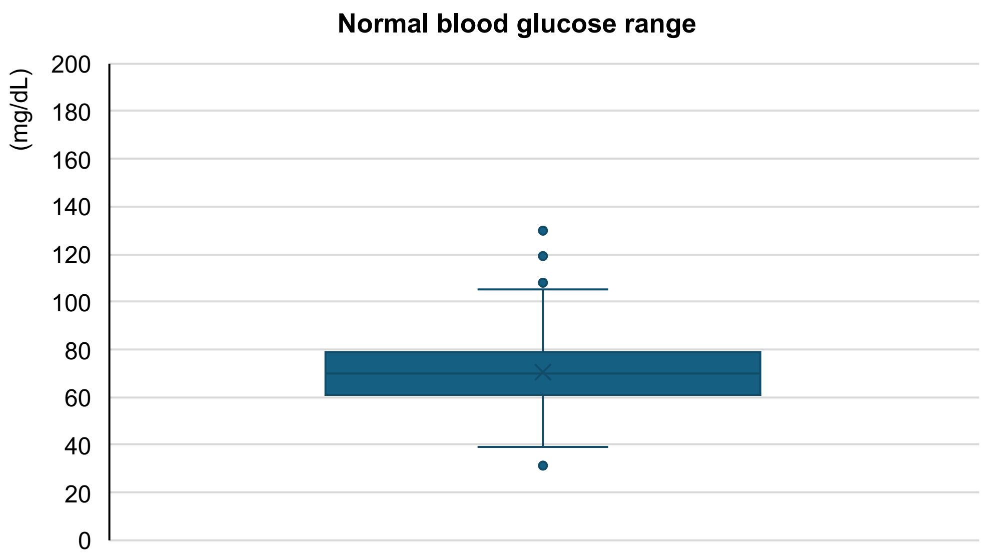
Normal blood glucose range
(mg/dL)
200
180
160
140
120
100
80
60
40
20
0

Figure S2

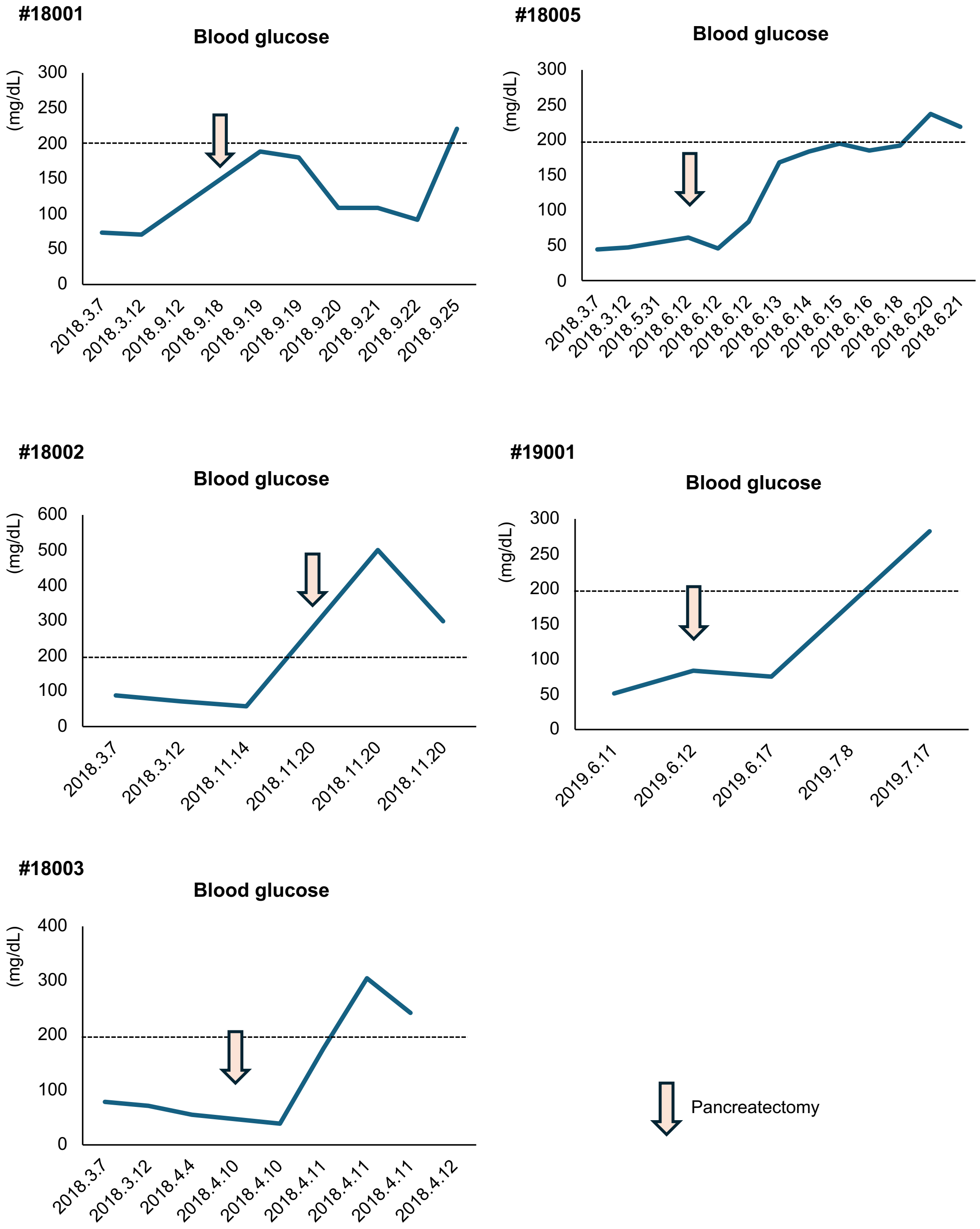

#18001
Blood glucose
(mg/dL)
300
250
200
150
100
50
0
2018.3.7
2018.3.12
2018.9.12
2018.9.18
2018.9.19
2018.9.19
2018.9.20
2018.9.21
2018.9.22
2018.9.25
#18005
Blood glucose
(mg/dL)
300
250
200
150
100
50
0
2018.3.7
2018.3.12
2018.5.31
2018.6.12
2018.6.12
2018.6.12
2018.6.13
2018.6.14
2018.6.15
2018.6.16
2018.6.18
2018.6.20
2018.6.21
#18002
Blood glucose
(mg/dL)
600
500
400
300
200
100
0
2018.3.7
2018.3.12
2018.11.14
2018.11.20
2018.11.20
2018.11.20
#19001
Blood glucose
(mg/dL)
300
250
200
150
100
50
0
2019.6.11
2019.6.12
2019.6.17
2019.7.8
2019.7.17
#18003
Blood glucose
(mg/dL)
400
300
200
100
0
2018.3.7
2018.3.12
2018.4.4
2018.4.10
2018.4.10
2018.4.11
2018.4.11
2018.4.11
2018.4.12
Pancreatectomy

Figure S3

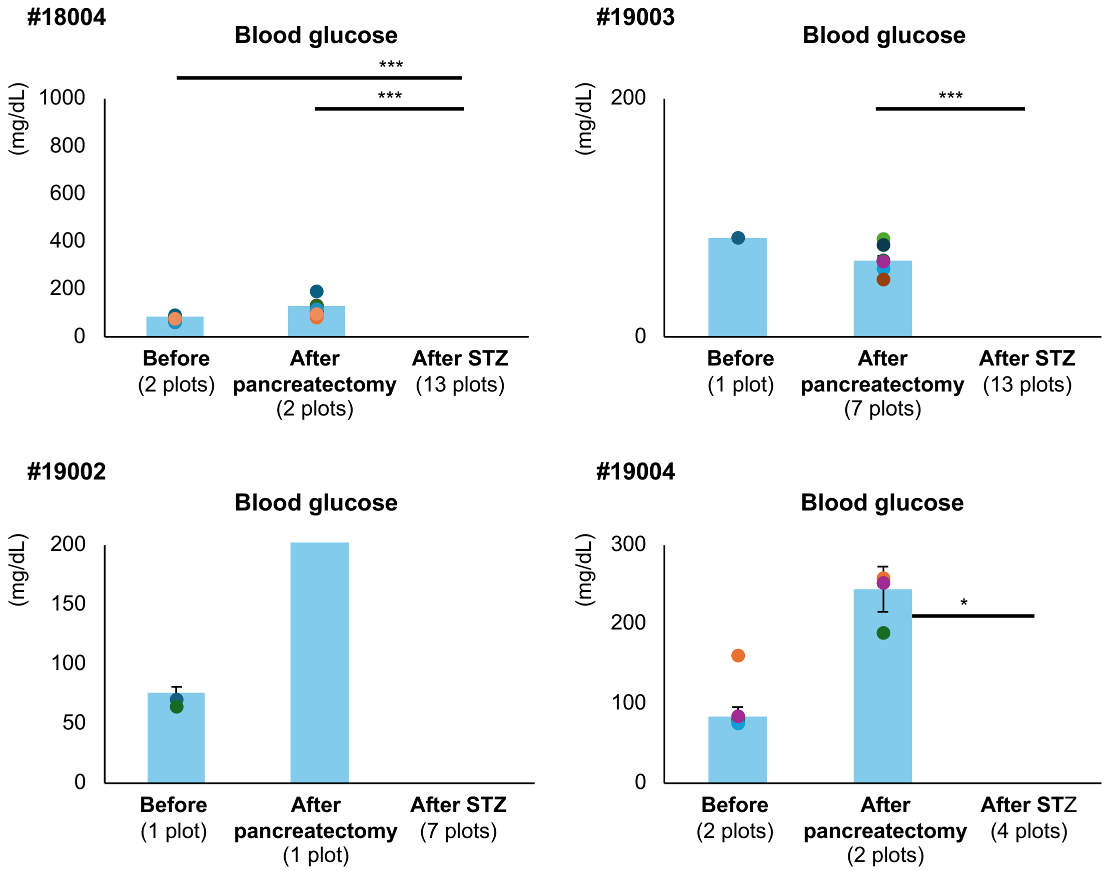
#18004
Blood glucose
(mg/dL)
1000
800
600
400
200
0
***
***
Before
(2 plots)
After
pancreatectomy
(2 plots)
After STZ
(13 plots)
#19003
Blood glucose
(mg/dL)
200
0
***
Before
(1 plot)
After
pancreatectomy
(7 plots)
After STZ
(13 plots)
#19002
Blood glucose
(mg/dL)
200
150
100
50
0
Before
(1 plot)
After
pancreatectomy
(1 plot)
After STZ
(7 plots)
#19004
Blood glucose
(mg/dL)
300
200
100
0
*
Before
(2 plots)
After
pancreatectomy
(2 plots)
After STZ
(4 plots)

Figure S4

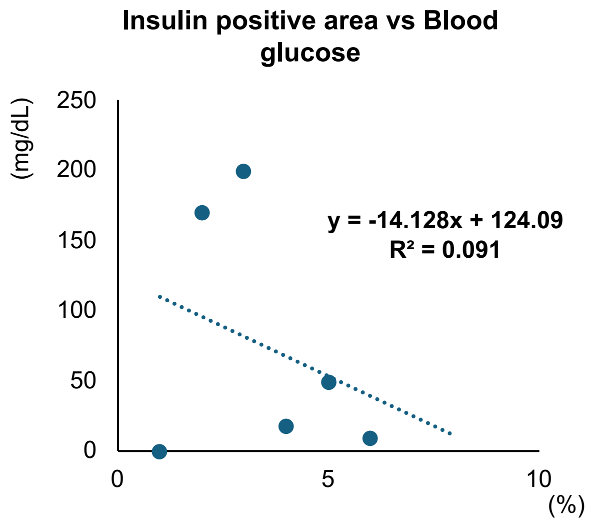

Insulin positive area vs Blood glucose
(mg/dL)
250
200
150
100
50
0
0
5
10
(%)
y = -14.128x + 124.09
R² = 0.091

Figure S5

#19005: STZ 100 mg/kg

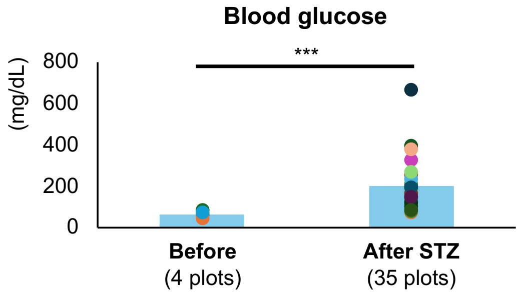

#21003: STZ 50 mg/kg 2 times

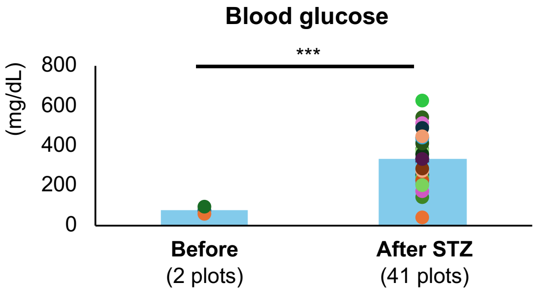

#19006: STZ 100 mg/kg

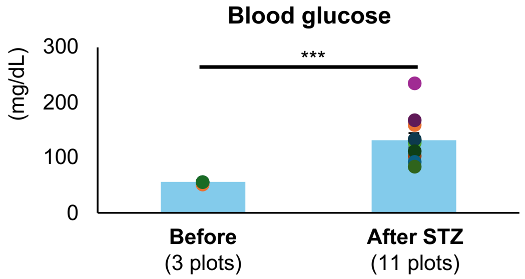

#21004: STZ 50 mg/kg

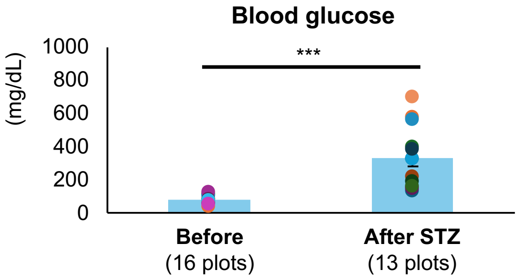

#19007: STZ 100 mg/kg

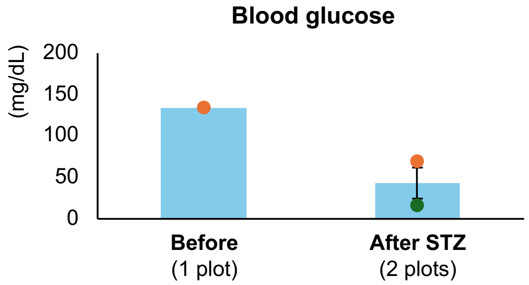

#23002: STZ 50, 60, 80 mg/kg

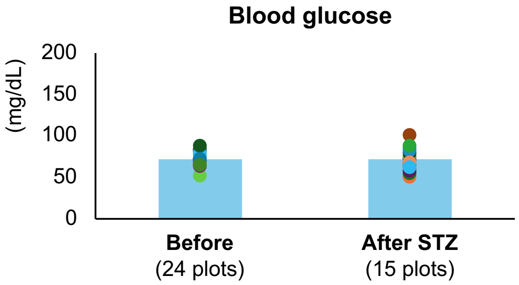

#19008: STZ 110 mg/kg

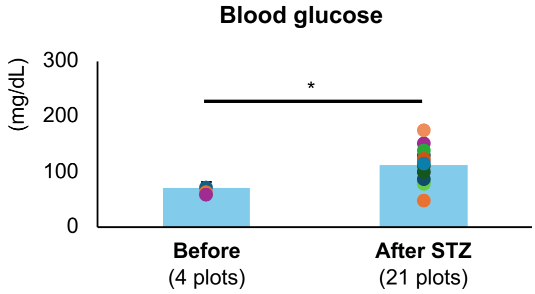

Figure S6

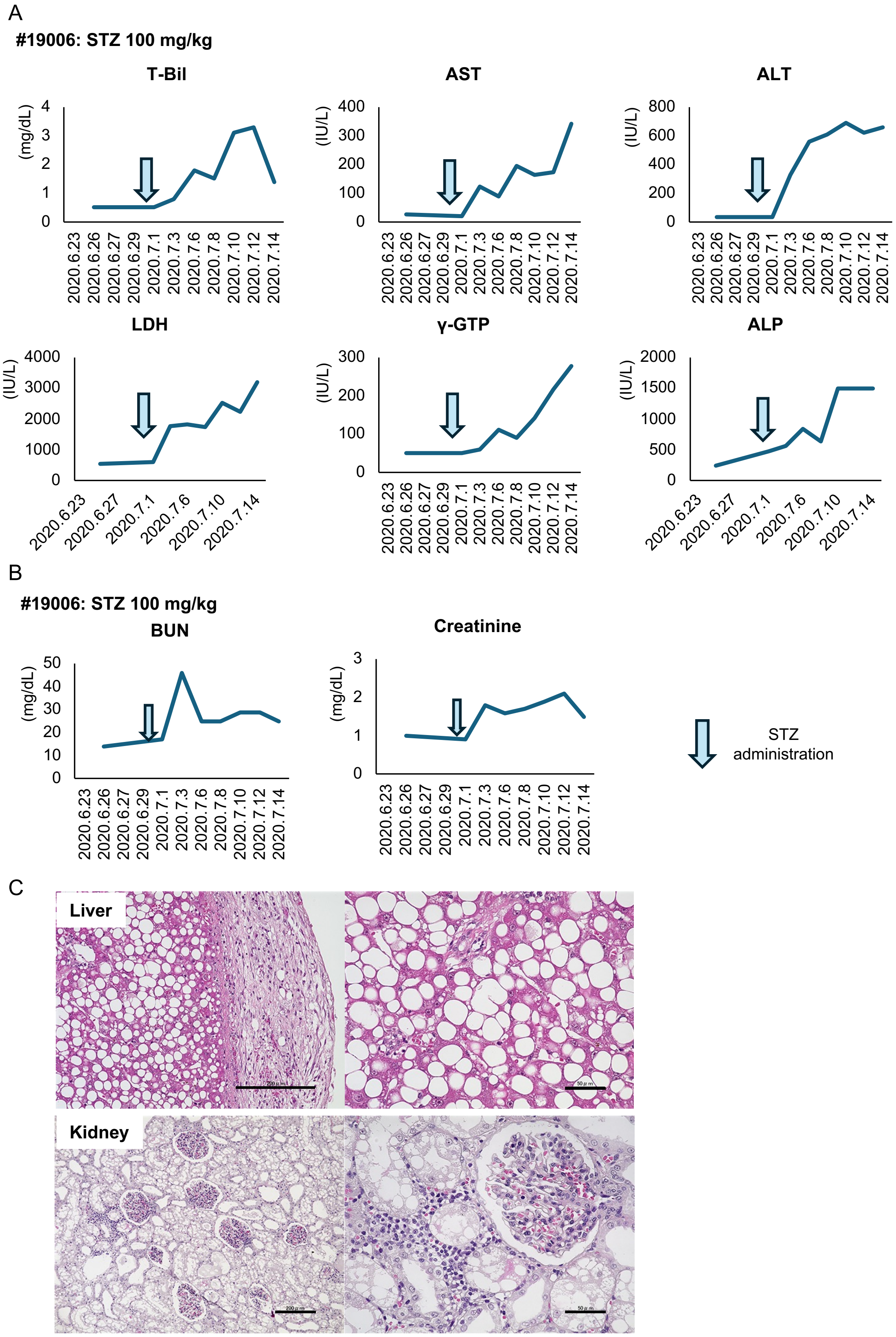
A
#19006: STZ 100 mg/kg
T-Bil
(mg/dL)
AST
(IU/L)
ALT
(IU/L)
LDH
(IU/L)
γ-GTP
(IU/L)
ALP
(IU/L)
B
#19006: STZ 100 mg/kg
BUN
(mg/dL)
Creatinine
(mg/dL)
STZ administration
C
Liver
Kidney

Figure S7

#24001: STZ 20 mg/kg × 5 days, 2 times

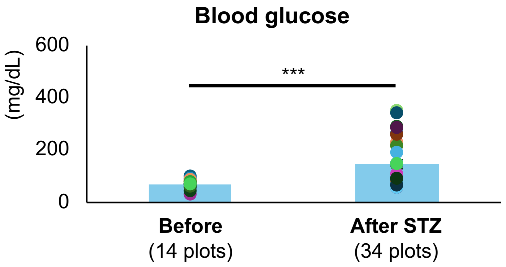

#24002:
STZ 30, 20, 20, 30, 30, 40, 50 mg/kg × 5 days, respectively

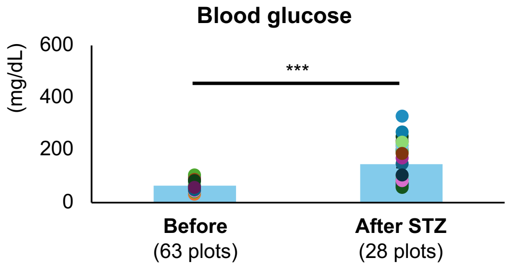

#25002: STZ 20 mg/kg × 5 days, 4 times

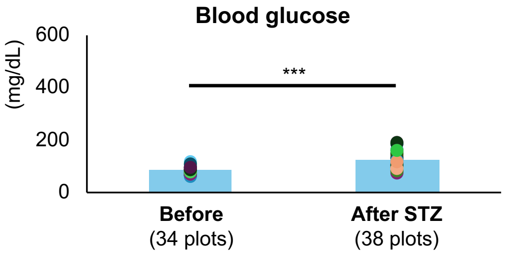

Figure S8

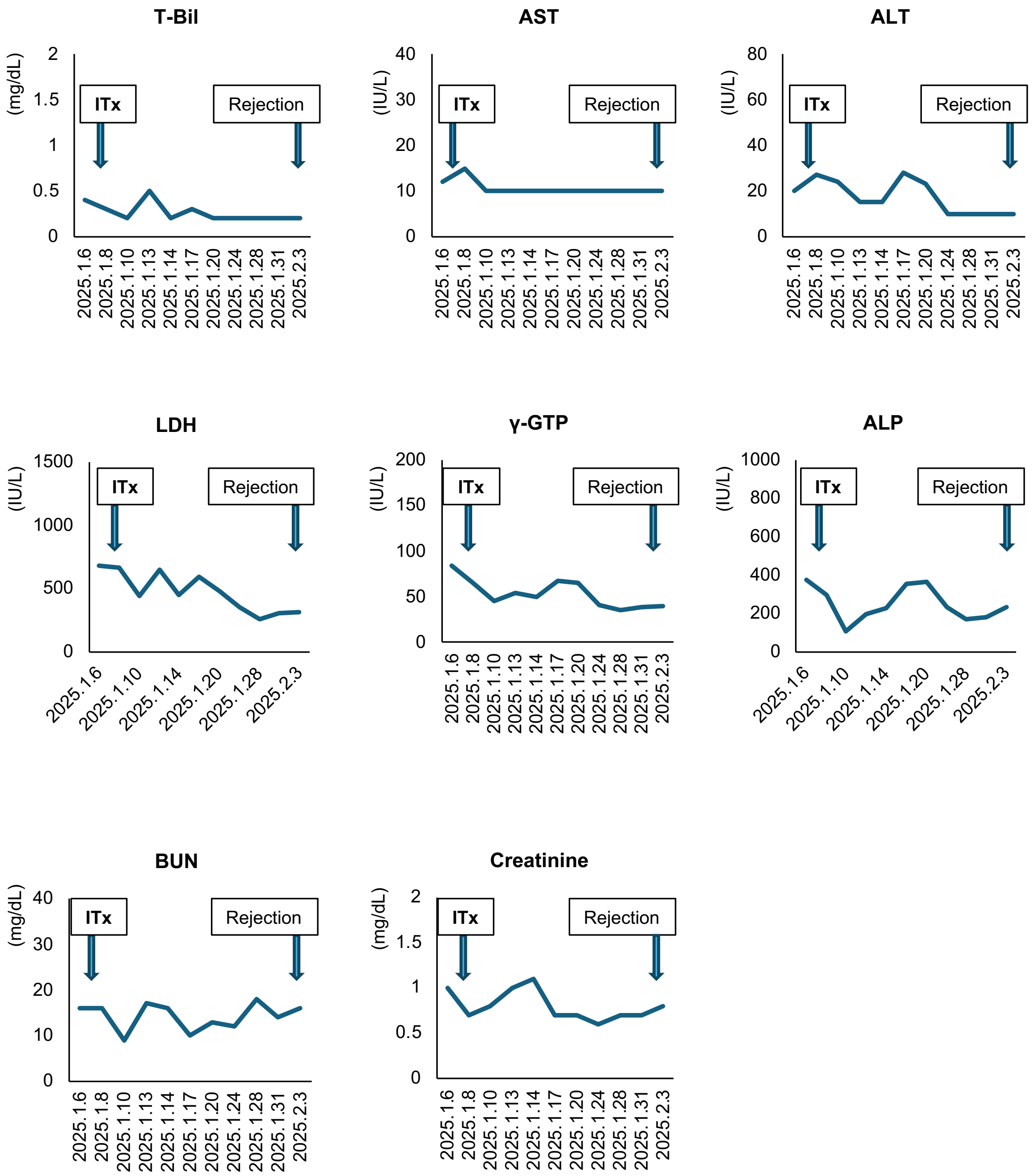
T-Bil
(mg/dL)
2
1.5
1
0.5
0
ITx
Rejection
2025.1.6
2025.1.8
2025.1.10
2025.1.13
2025.1.14
2025.1.17
2025.1.20
2025.1.24
2025.1.28
2025.1.31
2025.2.3
AST
(IU/L)
40
30
20
10
0
ALT
(IU/L)
80
60
40
20
0
LDH
(IU/L)
1500
1000
500
0
γ-GTP
200
150
100
50
ALP
1000
800
600
400
200
BUN
Creatinine

Figure S9

A

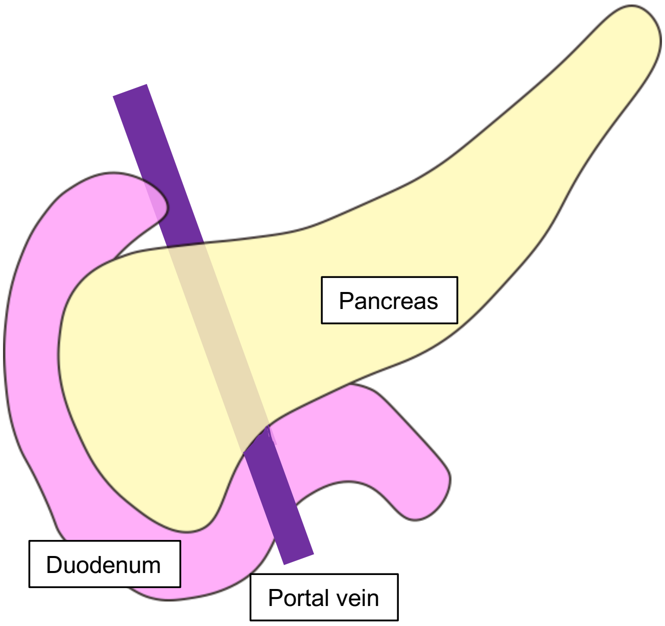

Pancreas
Duodenum
Portal vein

B

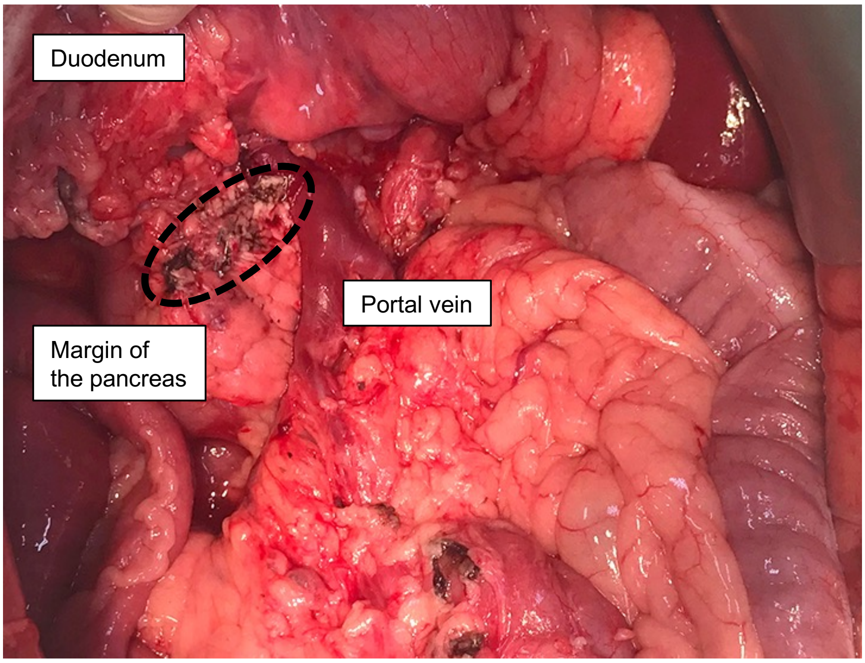

Duodenum
Portal vein
Margin of
the pancreas

Figure legends

Figure S1. Normal blood glucose range of the Japanese macaque

Blood glucose data from 23 healthy Japanese macaques who received no treatments. There were 139 blood glucose plots, and the 95% confidence interval of the data was defined as the normal blood glucose range of the Japanese macaque.

Figure S2. Changes in blood glucose levels before and after induction of diabetes in Model 1

Five monkeys (#18001, #18002, #18003, #18005, and #19001) underwent pancreatectomy (arrows). Achievement of diabetes was defined as blood glucose > 200 mg/dL.

Figure S3. Changes in blood glucose levels before and after induction of diabetes in Model 2

Mean blood glucose levels before and after pancreatectomy, and after STZ in each monkey (#18004, #19002, #19003, and #19004). The data are presented as mean ± SEM. *: $p < 0.05$, ***: $p <0.001$

STZ, streptozotocin; SEM, standard error of the mean

Figure S4. Regression analysis between the ratio of the insulin-positive area per islet and mean blood glucose level in Model 2

Correlation between the ration of the insulin-positive area per islet (X-axis) and mean blood glucose level (Y-axis) was assessed using regression analysis. $R^2>0.7$ is considered as strong correlation between the two parameters.

Figure S5. Changes in blood glucose levels before and after induction of diabetes in Model 3

Mean blood glucose levels before and after STZ in each monkey (#19005, #19006, #19007, #19008, #21003, #21004, and #23002). The data are presented as mean ± SEM. *: $p < 0.05$, ***: $p < 0.001$.

Figure S6. Changes in hepatic and renal functional parameters in monkey #19006 before and after STZ administration

A. The hepatic parameters comprised T-Bil, AST, ALT, LDH, γ-GTP, and ALP. B. The renal parameters comprised BUN and creatinine. The blue arrow indicates the timing of the STZ injections. C. HE images of the recovered liver (upper) and kidney (lower). The original size of the scale bar is 200 μm (left images) and 50 μm (right images).

STZ, streptozotocin; T-Bil, total bilirubin; AST, aspartate aminotransferase; ALT, alanine aminotransferase; LDH, lactate dehydrogenase; γ-GTP, γ-glutamyltransferase; ALP, alkaline phosphatase; BUN, blood urea nitrogen; HE, hematoxylin and eosin

Figure S7. Changes in blood glucose levels before and after induction of diabetes in Model 4

Mean blood glucose levels before and after STZ in each monkey (#24001, #24002 and #25002). The data are presented as mean ± SEM. ***: $p < 0.001$.

STZ, streptozotocin; SEM, standard error of the mean

Figure S8. Changes in hepatic and renal functional parameters in monkey #24001

during the postoperative period

T-Bil, AST, ALT, LDH, γ-GTP, and ALP (hepatic parameters), and BUN and creatinine (renal parameters) were monitored for 26 days.

T-Bil, total bilirubin; AST, aspartate aminotransferase; ALT, alanine aminotransferase; LDH, lactate dehydrogenase; γ-GTP, γ-glutamyltransferase; ALP, alkaline phosphatase; BUN, blood urea nitrogen

Figure S9. Scheme of subtotal pancreatectomy (located in the supplemental file)

A. Diagram showing the resection line during subtotal pancreatectomy. B. Intraoperative image after pancreatectomy.
